# Effect of a 2D-Modification of Cs_2_AgBiBr_6_ on Nucleation and Contact Formation of Subsequently Deposited Hole Transport Layers as Revealed by *In Situ* Growth Studies

**DOI:** 10.1021/acsami.5c24299

**Published:** 2026-01-30

**Authors:** Tim P. Schneider, Fabian Schmitz, Teresa Gatti, Derck Schlettwein

**Affiliations:** † Institute of Applied Physics, Justus Liebig University Giessen, Heinrich-Buff-Ring 16, 35392 Gießen, Germany; ‡ Center of Materials Research (ZfM), Justus Liebig University Giessen, Heinrich-Buff-Ring 16, 35392 Gießen, Germany; § Department of Applied Science and Technology, 19032 Politecnico di Torino, Corso Duca degli Abruzzi 24, 10129 Torino, Italy

**Keywords:** double perovskite, Cs_2_AgBiBr_6_, 2D perovskite, thin film growth, copper phthalocyanine, pentacene

## Abstract

A two-dimensional (2D) perovskite interlayer prepared by modification of a three-dimensional (3D) perovskite absorber with organic ammonium ions such as butylammonium (BA^+^) or phenethylammonium (PEA^+^) between the 3D perovskite and contact layers is widely known to significantly improve the performance of perovskite solar cells. This has also been confirmed previously for the lead-free double perovskite absorber Cs_2_AgBiBr_6_. In this work, film growth of copper phthalocyanine (CuPc) or pentacene (Pn), used as model hole transport materials (HTM), was investigated. Mimicking solar cell geometry, the HTMs were evaporated onto thin films of 2D perovskites BA_4_AgBiBr_8_ or PEA_4_AgBiBr_8_, as well as on 3D Cs_2_AgBiBr_6_, either in its pristine form or after modification by BA^+^ or PEA^+^. The morphology and work function were inspected intermittently with respect to the evaporation of the HTMs by Kelvin probe force microscopy at different average film thicknesses. By these means, the origin of device improvements following a 2D-modification in contact with HTMs, as established earlier, was revealed by analyzing in detail the interface of the HTM with the respective perovskite starting at monolayer coverage and proceeding toward bulk thickness. On modified Cs_2_AgBiBr_6_, the energy alignment between the perovskite and the HTM was found to be well confined, and the growth of both HTMs was improved compared to pristine Cs_2_AgBiBr_6_. HTM growth occurred more homogeneously and led to layer formation, even at early stages of deposition. For CuPc as HTM, these changes were accompanied by preferential formation of needles in a crystal phase different from that formed on pristine Cs_2_AgBiBr_6_, as also detected on 2D PEA_4_AgBiBr_8_. Pn formed large dendritic islands on the 2D perovskites as well as on layered terraces formed upon ammonium modification of Cs_2_AgBiBr_6_, in contrast to the growth of small grains on pristine Cs_2_AgBiBr_6_. Implications of these observed changes in film growth and energy level alignment on the observed contact characteristics with the HTMs in model solar cells are discussed. Insight into the mechanism of improving perovskite-based devices by use of 2D/3D perovskite heterostructures is, thereby, provided by these measurements using CuPc or Pn as model HTMs.

## Graphical abstract

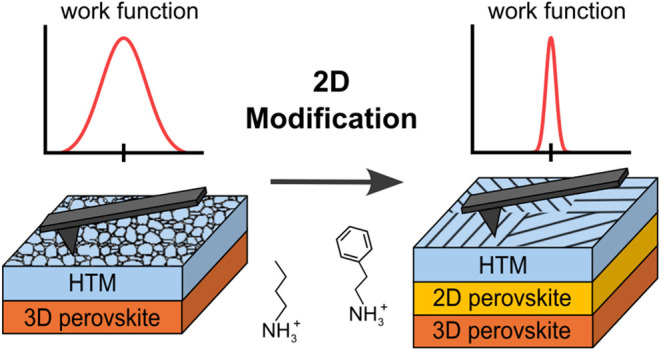

## 1 Introduction

Photovoltaic cells have become a key player in dealing with the world’s increasing energy demand. Solar cells using lead-based perovskite absorbers have achieved efficiencies comparable to silicon,^1^ but have to deal with chemical instability, toxicity, and as a consequence, environmental issues.^2–5^ Lead-free counterparts represent an attractive alternative since they allow us to circumvent these problems. Therefore, based on its decent air-stability, the lead-free halide double perovskite Cs_2_AgBiBr_6_ (Figure 1(a)), e.g., gained prominent interest and was studied in the context of devices such as X-ray-^6^ or photodetectors,^7,8^ memristors,^9,10^ and also solar cells.^11,12^ Although the overall performance of the latter is still not comparable to lead-based perovskite absorbers,^12^ their cell efficiency could recently be increased by employing a two-dimensional (2D)-modification of Cs_2_AgBiBr_6_, which significantly improved the contact to the hole transport layer.^13,14^

**Figure 1:**
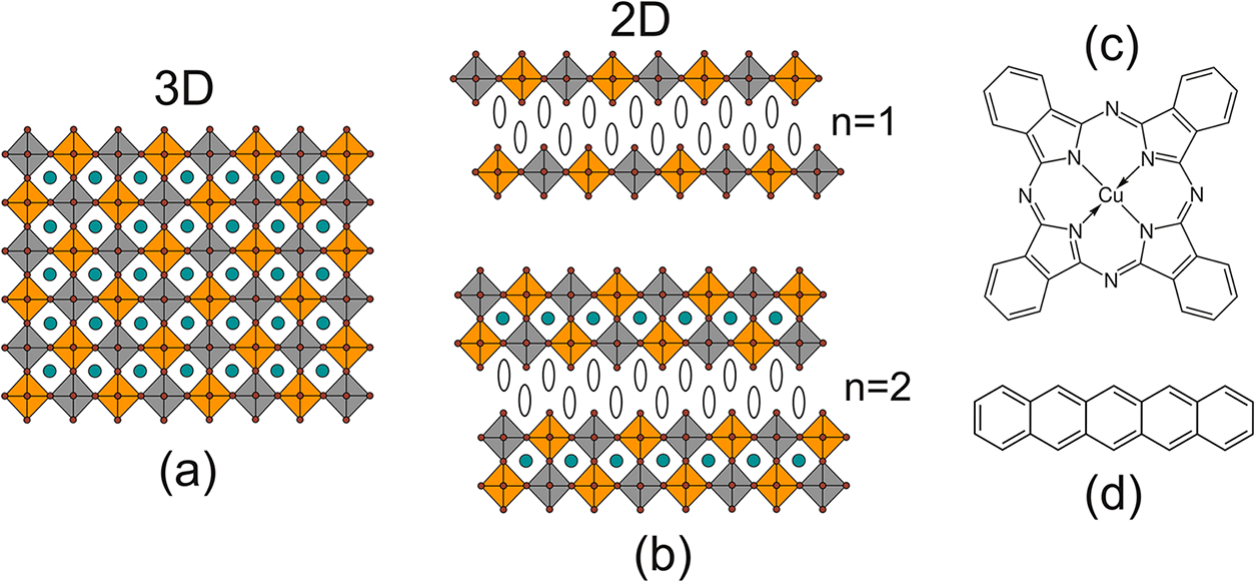
Scheme of the crystal structure of Cs_2_AgBiBr_6_ (a), a three-dimensional network of alternating [AgBr_6_] (gray) and [BiBr_6_] (orange) octahedra with Cs^+^ ions (turquoise) filling the gaps in between, and (b), corresponding 2D perovskite phases characterized by a restricted octahedral network due to incorporation of spacer cations, exemplarily shown for one (*n* = 1) or two (*n* = 2) octahedral layers between the spacers. Structural formulas of the model HTMs are shown (c) for copper phthalocyanine (CuPc) and (d) for pentacene (Pn).

In 2D perovskite phases, the symmetry of the three-dimensional (3D) octahedral network is broken along one crystal direction, caused by the substitution of the A-cations in perovskites (ABX_3_) by large organic spacer cations (A_2_B′B″X_6_ for double perovskites) that are too large to fit into the octahedral network.^15,16^ The resulting phase with only one octahedral layer (*n* = 1) between each layer of spacer cations is generally called 2D, whereas phases with two or more octahedral layers (*n* ≥ 2) between the spacer layers are called quasi-2D.^15,16^ The structure of such phases is shown in Figure 1(b) for a substitution of Cs in Cs_2_AgBiBr_6_ by alkylammonium bromides. In comparison to the 3D phase, these different 2D phases showed further enhanced environmental stability and altered optical and electrical properties, depending on the respective cation.^15–17^ By now, different approaches to combine different 2D and 3D phases have been reported. The mixture of different phases within a given film, as well as 2D/3D heterostructures, which consist of an underlying 3D phase with different 2D and/or quasi-2D phases on top, became of interest due to successful defect passivation and enhanced tolerance against moisture and oxygen.^15–18^ 2D/3D heterostructures can be realized by vapor deposition,^19,20^ mechanical pressing,^21,22^ or by the more facile route of just applying a spacer cation salt solution on top of an already prepared 3D phase.^13,14,23,24^ In this case, the salt reacts with the surface, and different 2D phases are formed. Recently, it was found that the different phases were not distributed evenly but that they were preferentially formed at grain boundaries of the 3D phase.^24^

Different 2D counterparts of Cs_2_AgBiBr_6_ have already been investigated, especially BA_4_AgBiBr_8_ ^25,26^ and PEA_4_AgBiBr_8_,^26^ containing the spacer cations butylammonium (BA^+^) or phenethylammonium (PEA^+^), by which the network of alternating [AgBr_6_] and [BiBr_6_] octahedra is restricted to 2D layers separated by the respective spacer cations (Figure 1(b), *n* = 1). Phase-pure films are easily obtained by spin-coating a stochiometric solution of AgBr, BiBr_3_, and BABr or PEABr, or by spin-coating a solution of presynthesized BA_4_AgBiBr_8_ or PEA_4_AgBiBr_8_.^25–27^ 2D/3D heterostructures were directly obtained by spin-coating solutions of BABr^13^ or PEABr^14^ on top of preformed Cs_2_AgBiBr_6_ films and earlier studies proved successful implementation in solar cells.^13,14^ Their improved performance over cells of pristine Cs_2_AgBiBr_6_ was ascribed to enhanced contact selectivity and hole extraction.^13,14^

In this work, growth studies of the organic hole transport materials (HTM) copper phthalocyanine (CuPc, Figure 1(c)) and pentacene (Pn, Figure 1(d)) on 2D, 3D and 2D/3D heterostructured silver bismuth bromide double perovskite films were conducted to reveal the origin of the reported device improvements when using 2D/3D heterostructures. Characteristic changes in contact formation to the HTMs were observed for both of these HTMs on the different double perovskite substrates. CuPc and Pn were chosen due to their vacuum-processability,^28–31^ which is necessary to conduct *in situ* growth studies. Even though most common and best performing HTMs are solution-processed, evaporated CuPc and Pn have been successfully applied to perovskite solar cells. CuPc is well-known as a molecular semiconductor and has already been used in perovskite solar cells as a hole conductor,^29,32–35^ as an additive for the perovskite precursor,^36,37^ and as a buffer layer in tandem cells.^38^ Related phthalocyanine materials with substitutions on the ring^32,39,40^ or containing other central metals^41^ have been investigated as contact layers as well. Pn, aside from a prominent interest in its use in thin film transistors,^30,42,43^ has already been successfully studied as an HTM in perovskite solar cells.^31,44–46^ Further, the substituted version TIPS-pentacene was used to absorb excess photon energy due to singlet fission and, thereby, enhanced the photoconversion efficiency of perovskite solar cells.^47–49^ For both CuPc and Pn, vapor deposition onto different substrates has been widely studied and different polymorphic phases are well-known in the literature.^28,30,50–58^ Contact formation to other perovskite absorbers like MAPbI_3_ ^45,59^ and FASnI_3_ ^60^ has been reported to which the present results are directly referenced. We will study the morphology and work function of these HTMs at different average film thicknesses intermittently during their deposition on the respective 2D or 3D double perovskite films. The crystal structure of CuPc and Pn deposited on such different films was independently investigated by (*ex situ*) X-ray diffraction and (*in*- and *ex situ*) ultraviolet–visible (UV–vis) transmission spectroscopy. Consequences of the observed differences in HTM growth for contact characteristics are shown in model devices, and pathways toward further optimization will be discussed.

## 2 Experimental Section

### 2.1 Sample Preparation

Glass substrates coated with fluorine-doped tin oxide (FTO, Sigma-Aldrich, ∼13 Ω/sq) or uncoated glass substrates (Thermo Scientific) were subsequently ultrasonicated for 15 min each in an aqueous detergent solution (Roth RBS 25), acetone (Roth, ≥99.5%), and isopropanol (Roth, ≥99.5%). FTO substrates were then treated by UV/ozone for 10 min prior to deposition of a compact TiO_2_ layer by spin-coating a commercial solution (Solaronix Ti-Nanoxide BL/SC) at 4000 rpm for 30 s followed by annealing at 500 °C for 1 h in air. Before depositing any perovskite layer, another UV/ozone treatment was performed and the samples were immediately transferred to a nitrogen-filled glovebox (<1 ppm of O_2_, <1 ppm of H_2_O).

To prepare 2D layered double perovskites, AgBr (Alfa Aesar, 99.5%), BiBr_3_ (Thermo Scientific, 99%) and BABr (butylammonium bromide, TCI, >98%) or PEABr (phenethylammonium bromide, Greatcell Solar Materials) were dissolved at a molar ratio of 1:1:4 in a mixture of 4:1 dimethylformamide (extra-dry, Thermo Scientific, 99.8%) and dimethylsulfoxide (anhydrous, Thermo Scientific, 99.8%) achieving a composition corresponding to 0.25 mol/L BA_4_AgBiBr_8_ or PEA_4_AgBiBr_8_, respectively, after stirring overnight at 75 °C. Films were obtained by spin-coating these solutions onto TiO_2_-coated FTO glass or uncoated glass for 40 s at 4000 rpm with an acceleration time of 20 s, followed by annealing at 100 °C for 4 min.

To prepare 3D Cs_2_AgBiBr_6_ double perovskite, AgBr, BiBr_3_, and CsBr (Fluorochem, >99%) were dissolved at a molar ratio of 1:1:2 in dimethylsulfoxide, yielding a 0.5 mol/L solution after stirring overnight at 75 °C, which was then spin-coated onto TiO_2_-coated FTO glass or uncoated glass for 40 s at 4000 rpm with an acceleration time of 3 s. The films were annealed at 285 °C for 5 min afterward. The samples were allowed to cool before further treatment by spin coating a 0.05 mol/L solution of BABr or PEABr in isopropanol (extra-dry, Acros Organics, 99.8%) on top of the Cs_2_AgBiBr_6_ samples using the same parameters and allowed to dry without further annealing.

All samples containing different perovskite films on TiO_2_-coated FTO glass were transferred to a vacuum chamber (≈5 × 10^–7^ mbar) equipped with an atomic force microscope (AIST-NT, VacuScope 1000) and a physical vapor deposition system. CuPc (Sigma-Aldrich, >99.95%) or Pn (Sigma-Aldrich, >99.9%) were evaporated from resistively heated boron nitride crucibles while monitoring the deposition rate and film thickness with a calibrated quartz microbalance, using a deposition rate between 0.3–0.5 nm/min. Evaporation was stopped when the measured work function reached a constant value. Samples containing different perovskite films on glass were transferred to another vacuum chamber (≈ 2 × 10^–6^ mbar) equipped with a comparable physical vapor deposition system and optical feedthrough enabling *in situ* UV–vis spectroscopy in transmission during film growth of CuPc on the respective perovskite film.

For achieving model devices in a solar cell architecture, FTO substrates were partially etched prior to TiO_2_ deposition, followed by the same procedures as described above for preparation and modification of Cs_2_AgBiBr_6_. Then, 70 nm of CuPc or Pn were evaporated in a vacuum chamber (≈5 × 10^–7^ mbar) on top of the perovskite layer without breaking the inert atmosphere. Finally, 60 nm thick gold electrodes were deposited by electron-beam evaporation (Leybold Univex 300).

### 2.2 Measurements

The morphology and contact potential difference between the sample and the tip were scanned before and in between stepwise deposition of the HTMs. Measurements were performed in noncontact two-pass frequency-modulated Kelvin probe force microscopy with an amplitude of 20 nm and a sampling rate of 0.5 line/s using conducting Spark 350 Pt (length 125 μm) probes from NuNano. In the first pass, the morphology was obtained. The contact potential difference was measured in the second pass after lifting the probe by 10 nm and applying 3 V AC. The surface work function was then calculated after calibrating the work function of the probe to freshly cleaved highly oriented pyrolytic graphite (HOPG, MikroMasch, grade ZYA) with the known work function of (4.60 ± 0.03) eV.^61^ Reference measurements were obtained before and after each measurement at a given film thickness. Atomic force microscopy (AFM) images were evaluated using Gwyddion software; in particular, the watershed algorithm was used for masking the grains.

X-ray diffraction (XRD) was conducted with a Rigaku SmartLab using CuKa-radiation at a grazing incidence of 1° and a scan speed of 5°/min. UV–vis spectra were recorded in transmission with a Tec5 spectrometer in ambient air for samples prepared during *in situ* work function measurements or in vacuum during film growth. Optical microscopy images were taken with a Keyence VK-9710K confocal laser microscope. Current–voltage characteristics of the samples were measured in air with an IviumStat potentiostat from Ivium Technologies at a rate of 0.05 V s^–1^. A xenon arc lamp (LS0106, LOT Oriel) was used for illuminating 16 mm^2^ cells using a slightly larger 25 mm^2^ shadow mask with AM1.5 white light at a power density of 100 mW cm^–2^ established by the use of a calibrated ML-020VM pyranometer (EKO Instruments).

## 3 Results and Discussion

All double perovskite films used as a substrate for the deposition of the organic hole transport materials were prepared on a compact n-TiO_2_ layer on a FTO-coated glass. Thereby, a geometry as close as possible to n-i-p solar cell devices was achieved to perform the study of contact formation to the HTMs. First, film growth and contact formation are described on 2D double perovskite films BA_4_AgBiBr_8_ and PEA_4_AgBiBr_8_. Then, the growth on Cs_2_AgBiBr_6_ modified with 2D perovskites will be discussed.

### 3.1 Growth of CuPc or Pn on 2D Double Perovskite Model Surfaces

First, the growth of HTMs CuPc and Pn was studied on the pure 2D double perovskites BA_4_AgBiBr_8_ and PEA_4_AgBiBr_8_. Since typically rather small grains had been obtained in thin films,^26^ a solvent engineering approach with mixtures of DMF and DMSO^7,9,11,62^ was used to realize larger crystalline domains of BA_4_AgBiBr_8_ and PEA_4_AgBiBr_8_, preferred for the present growth studies, which are shown in Figure 2(a),(f) as AFM height images. Large micrometer-sized terraces were obtained with only a few vacancies and islands of adlayers. Large terraces with well-defined steps are formed. These steps are mostly parallel to each other, with some 90° angles visible, fitting well to the [100] and [010] low index directions of the cubic structure.

**Figure 2:**
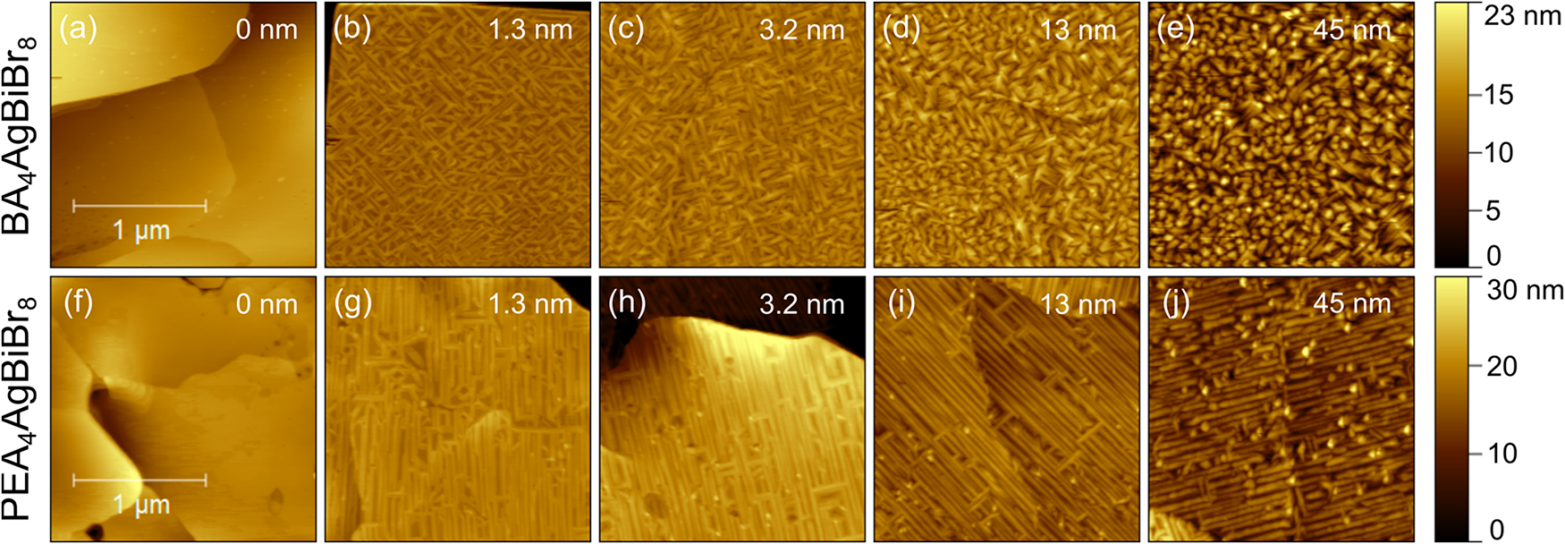
Morphology for different average film thicknesses of CuPc evaporated on top of BA_4_AgBiBr_8_ ((a)–(e)) and PEA_4_AgBiBr_8_ ((f)–(j)).

Growth of CuPc evaporated onto BA_4_AgBiBr_8_ is shown for different average film thicknesses of CuPc as AFM height images in Figure 2(b)–(e), and the corresponding work function images in Figure S1­(b)–(e) in the SI. Already after deposition of 1.3 nm (Figure 2(b)), a characteristic arrangement of CuPc was observed. Needles formed along certain directions vertical to each other, in good agreement with the steps on BA_4_AgBiBr_8_ terraces, leaving gaps of uncovered BA_4_AgBiBr_8_ between them. Upon further deposition (Figure 2(c)), the needles mainly became wider (about 25 nm) and closed the gaps between them. An underlying terrace step of the double perovskite can still be observed by a sequence of CuPc needles aligned along a curved line from the bottom left to the top middle of Figure 2(c). A preferential formation of the needles along step edges is, thereby, indicated. At 13 nm (Figure 2(d)), most crystals started to grow perpendicular to the surface whereas along defined lines (presumably again indicating a terrace step or grain boundary of the perovskite underneath) the needle-like appearance continued. Beyond 45 nm, a widely homogeneous morphology was obtained (Figure 2(e)) consisting of crystals with a widely random orientation. Consequently, the RMS roughness of the film significantly increased from 2.1 nm at 13 nm to 3.9 nm at 45 nm. By the observed film morphology, it is indicated that terrace steps of the 2D perovskite significantly affected the shape of CuPc grains up to a considerable film thickness. The work function shown in Figure S1­(a)–(e) was found to be very homogeneous for each film thickness despite prominent needle formation and was found to shift toward lower values. This is summarized in Figure 3 that shows the mean value of its distribution depending on film thickness. It saturated at 4.06 eV, which almost matches the value of 3.8–4.0 eV usually observed on other substrates like SiO_2_,^63^ Si(111)^64^ or phosphorene.^65^ *In situ* UV–vis absorption (Figure S12­(c),(d)) showed that CuPc nucleated in its α-phase on BA_4_AgBiBr_8_. Further, the spectra suggest an additional, albeit small, contribution of the β-phase when the average film thickness exceeded 20 nm. No reliable phase information could be obtained from XRD (Figure S11­(b)) due to the overlap of the CuPc and BA_4_AgBiBr_8_ signals. For the growth of phthalocyanines, preferential nucleation at step edges on different substrates was often observed.^66–70^ Also here, CuPc is arranged along layer steps of the underlying perovskite. Such an arrangement, however, was seen as well even on a given terrace without visible steps, presumably triggered by adlayers and vacancies that were seen on the uncovered terrace (Figure 2(a)) and speaking in favor of CuPc needle growth along the [100] and [010] substrate directions.

**Figure 3:**
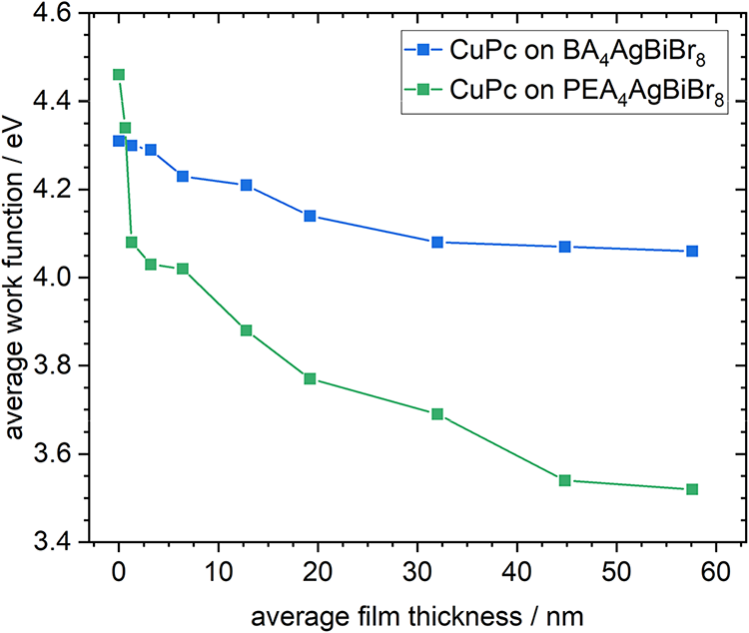
Work function depending on the average film thickness of CuPc on BA_4_AgBiBr_8_ or PEA_4_AgBiBr_8_.

Growth of CuPc on PEA_4_AgBiBr_8_, depicted in Figure 2(f)–(j), revealed similarities but also significant differences to its growth on BA_4_AgBiBr_8_. Again, after deposition of 1.3 nm (Figure 2(g)), needles of CuPc formed and were arranged along lines, roughly 26 nm wide and several hundreds of nanometers long, often even exceeding the scan width. Most of them were found to be oriented along a preferred direction parallel to each other, even crossing step edges on the PEA_4_AgBiBr_8_ terraces. An epitaxial relationship between the underlying PEA_4_AgBiBr_8_ and the growing crystals of CuPc is, thereby, indicated. Some structural defects on the underlying PEA_4_AgBiBr_8_ terraces like step edges, adatoms or vacancies may represent crystallization sites of the needles, but then the crystal lattice of PEA_4_AgBiBr_8_ seems to define the orientation of the needles, again presumably along the [100] and [010] substrate directions. Some needle orientation perpendicular to the preferred direction, in view of the cubic perovskite lattice, is in clear support of such a hypothesis. Very few round-shaped grains could be found as well, which are shaped like pillars growing perpendicular to the surface and sticking out about 20 nm high, presumably triggered by crystallization at ledge-sites and hence pointing in directions perpendicular to the terraces. As seen in Figure 2(h)–(j), such highly ordered growth was maintained during further deposition, even up to 45 nm average film thickness, however, accompanied by a decreased average length of the needles. The perovskite was fully covered from 13 nm, since then, the height difference from the top of a needle to a valley next to it was significantly smaller than the average film thickness (e.g., 2.1–3.6 nm in Figure 2(i) or 5.1–6.5 nm in Figure 2(j)). Therefore, the perovskite terraces are closely packed by CuPc needles. On a given perovskite grain, the needle direction was found constant, even across step edges, as described above. However, no lateral order can be expected for the orientation of different perovskite grains in films spin-cast on polycrystalline TiO_2_ on FTO on glass. Consistently, domains of differently oriented CuPc needles were found on adjacent grains, as seen, e.g., in the upper right corner of Figure 2(i) and in the lower right corner of Figure 2(j). The work function was again found very homogeneous for a given thickness of CuPc but decreased significantly faster already for the thinnest film compared to CuPc growth on BA_4_AgBiBr_8_ and continued to decrease until 45 nm (Figure 3) toward a considerably lower value of around 3.53 eV compared to literature^63–65^ and compared to the growth on BA_4_AgBiBr_8_. This difference could presumably be caused by the nucleation of CuPc in different crystal structures. Aside from the α-phase, significant additional contributions of the β- and η-phases were observed by *in situ* UV–vis spectroscopy, in particular at low CuPc film thickness on PEA_4_AgBiBr_8_ (Figure S12­(a),(b)). The formation of long needles (Figure 2(g)–(i)) fits well with the observed η-phase since this was previously found in nanowires.^52,53^ Although, at higher film thickness, the contribution of the η-phase to the spectra decreased relative to the α- and β-phases, which was accompanied by a shortening of the needles, the η-phase seemed to be still the dominant crystalline phase in the films with the highest crystal size and/or quality since it was the only phase detected in the corresponding XRD (Figure S11­(b)). As the size of the η-signal in the UV–vis spectra stayed widely constant for films exceeding 50 nm while the α- and β-signals increased, thicker films obviously nucleated mainly in the α- and β-phase of CuPc (Figure S12­(b)). The structure-directing influence of the surface toward crystallization of CuPc in the η-phase, therefore, faded with increasing CuPc thickness. To allow a more precise analysis of the needles, a 0.63 nm thin film of CuPc on PEA_4_AgBiBr_8_ was analyzed (Figure S3). Profiles were extracted and compared to the reported lattice constants of the different CuPc phases. In view of the uncertainties, the height of the lowest observed step of a needle roughly fitted to four times the (010) lattice constant either of the η-phase (0.377 nm)^52^ or of the α-phase (0.381 nm)^28,50,51^ and, hence, could be explained by a stack of four CuPc molecules with the (010)-plane parallel to the surface in either one of the two phases. A profile along a needle only showed uncorrelated noise of ±0.05 nm. Consequently, a given needle seems to consist of a single crystal rather than of a sequence of small individual grains, which would be in line with the η-phase exhibiting high crystallinity as detected by XRD. Consequently, the long axis of the needles is assumed to match the [001]-direction of the η-phase, reported as an axis of the observed nanowires.^52,53^

Since the two 2D double perovskites only differ in the organic spacer cations, the latter obviously cause the different growth mechanisms, the changes in work functions, and the formation of the different CuPc phases. The surface termination with BA^+^ or PEA^+^, respectively, led to significantly different conditions for adsorption, seed formation, and crystal growth in the initial stage of CuPc film growth.

Figure 4(a)–(e) show the morphology of Pn grown on BA_4_AgBiBr_8_. At an average film thickness of 2 nm (Figure 4(b)), large terraces with a height of around 2.2 nm above the BA_4_AgBiBr_8_ surface were formed. At some spots, up to 7 nm high islands began to grow on top of such terraces. As can be seen from the marked profiles (Figure S4, left), the steps of these islands can be explained by the reported spacing of 1.55 nm for Pn crystals in the thin film phase.^54,55^ A significant difference was observed for the Pn terraces, which must consist of two molecular layers but being too low, pointing toward a distortion of either Pn or BA molecules in the interface, which vanished upon formation of islands. A significant part of the substrate remained uncovered, corresponding to the average film thickness. A layered growth on these terraces was observed upon further deposition of Pn, leading to dendritic islands typical for Pn (Figure 4(c)).^30,55^ Molecules landing on the Pn terraces obviously led to height growth of these crystals, while molecules landing on the substrate between the islands led to lateral growth of the crystals since the gaps between the islands were almost closed at 10 nm, leading to the close packing of islands observed from 20 nm onward (Figure 4(d)). The observed step heights in Pn islands still fit to the interplanar spacing,^54,55^ revealed from the marked profiles (Figure S4, right). The observed discrepancies might be due to terraces that were too narrow to be measured adequately. The lateral grain sizes did not change further, but the roughness of the film increased from 6.9 nm at 20 nm to 10.1 nm at 70 nm (Figure 4(d),(e)), caused by the pyramidal crystal shape of the initially terraced seeds. Such growth mechanism of island formation was very similar to that typically observed for Pn grown on SiO_2_.^30,43,54–57^ XRD revealed the presence of Pn in the thin film phase, but cannot exclude its coexistence in the bulk phase (Figure S13­(b)). The morphology pattern was, in particular for higher film thickness, also seen in the measured work function (Figure S2­(b)–(e)). Appropriate masking of each height image led to a selection of only Pn islands, and the reversed mask selected the surface of the film between the islands (valleys). Applying these masks to the corresponding work function image allowed us to extract the work function of the two different regions separately. This procedure is illustrated schematically for two examples in Figure S5. Average values taken from these separate distributions are depicted in Figure 5. During deposition, the value found on top of Pn islands decreased faster than that measured in the valleys, which at the early stages of deposition mainly represented the substrate, as seen from the height images. Exceeding 20 nm of Pn, however, the decrease was slower and almost identical in both areas, indicating complete coverage of the perovskite by Pn. The work function in Pn valleys at 70 or 90 nm matches with the value measured on top of Pn islands at 20 or 30 nm, respectively. The actual film thickness in areas between the islands at the respective stage of deposition is, thereby, indicated since a similar thickness of Pn in peaks at 20 nm (30 nm) average film thickness and in valleys at 70 nm (90 nm) average film thickness can be assumed based on the work functions. For higher film thickness, the distribution of work function on top Pn islands became slightly asymmetric toward lower values (Figure S5­(b)), indicating the same effect: a lower work function for locally thicker Pn. The range of values matches well with the range of 3.9–4.1 eV found in literature for films of Pn on metals,^71^ ITO,^72^ or SiO_2_.^73^ This points out that BA^+^ did not significantly affect the growth of Pn compared with these other substrates.

**Figure 4:**
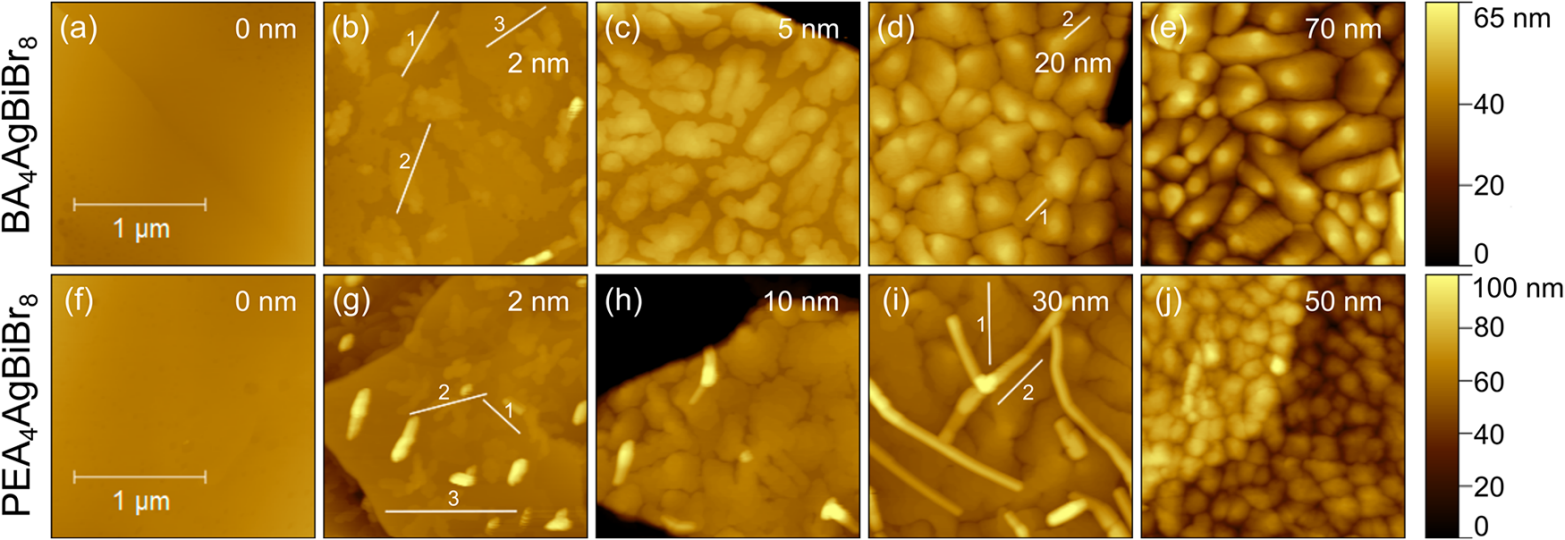
Morphology for different average film thicknesses of Pn evaporated on top of BA_4_AgBiBr_8_ ((a)–(e)) and PEA_4_AgBiBr_8_ ((f)–(j)).

**Figure 5:**
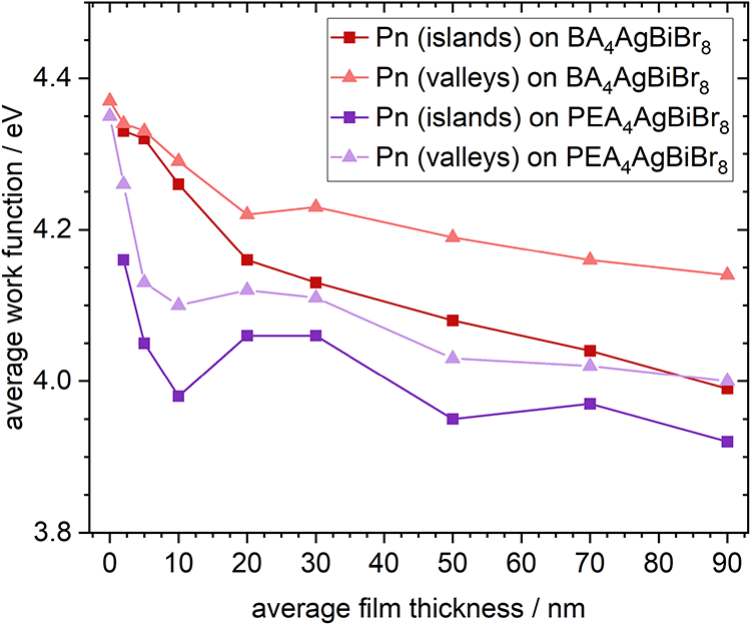
Work function depending on the average film thickness of Pn on BA_4_AgBiBr_8_ or PEA_4_AgBiBr_8_ found on top of Pn islands or in valleys between Pn islands.

Growth of Pn on PEA_4_AgBiBr_8_ showed many similarities but also some significant differences. The morphology of 2 nm of Pn evaporated onto PEA_4_AgBiBr_8_ (Figure 4(g)) showed well-defined dendritic islands. The lowest layer of a given island was found to fit one (profile 1) or two (profile 3) layers of Pn molecules corresponding to the literature (Figure S6, left),^54,55^ hence, a growth without any distortion at the interface in contact with PEA^+^ as opposed to BA^+^ was observed. Some islands already reached around 6 nm in height, consisting of four layers of Pn molecules (profile 2). A few elongated grains were sticking out of the surface by more than 40 nm. Upon further evaporation, the terraced islands grew similarly to those observed on BA_4_AgBiBr_8_, leaving gaps between islands even for 10 nm of Pn. Until 30 nm average film thickness, the additional elongated grains developed toward long connected filaments, as seen clearly in Figure 4(i). Such features were previously assigned to the bulk phase of Pn.^54^ Simultaneous nucleation in both phases was also discovered before on SiO_2_ at elevated temperatures.^58^ The step profiles in Figure 4(i) matched to the Pn thin film phase with an interplanar spacing of 1.55 nm (Figure S6, right),^54,55^ which is reported in literature for such morphology.^54^ A different type of growth was observed when an average film thickness of 30 nm Pn was exceeded. The morphology changed to significantly smaller grains (Figure 4(j)). One may speculate that the parallel growth of Pn in either the thin film or bulk structure led to rather disordered growth and such smaller grains. Unfortunately, XRD could not help to resolve this question since the Pn signals were superimposed to the very intense perovskite signals (Figure S13­(b)). Correlation of the work function and morphology was done in the same way as described above for Pn on BA_4_AgBiBr_8_, obtaining values characteristic for either the Pn islands or the areas between them, substrate, or, later, thinner layers of Pn. As seen in Figure 5, the work function decreased more rapidly on both sites compared to growth on BA_4_AgBiBr_8_ during the first stages of deposition and ended up being slightly lower. The difference in work function between islands and valleys at a higher average film thickness is found to be smaller than that for Pn on BA_4_AgBiBr_8_, in line with a more even morphology on PEA_4_AgBiBr_8_. At 90 nm, the work function found in Pn valleys matched with the value found on top Pn islands at 5–30 nm. It is, thereby, confirmed that Pn covers the entire perovskite surface. The work function of Pn on peaks and in valleys was in good agreement with literature.^71–73^

Comparing the results obtained for the two contact materials CuPc and Pn, it becomes clear that the PEA^+^ spacer cation obviously affected the film growth quite differently than did BA^+^. For both CuPc and Pn, the films grown on PEA_4_AgBiBr_8_ showed a lower work function. CuPc also nucleated differently with the more common α- and β-phases on BA_4_AgBiBr_8_ as opposed to the rather unusual η-phase on PEA_4_AgBiBr_8_, accompanied by the growth of elongated needles. The shape of the Pn islands was dendrite-like for thin films on both BA_4_AgBiBr_8_ or PEA_4_AgBiBr_8_. On BA_4_AgBiBr_8_ these islands continued to grow for thicker films while on PEA_4_AgBiBr_8_ a larger number of smaller grains was detected. The intermediate formation of additional Pn filaments was found to be more prominent on PEA_4_AgBiBr_8_ than on BA_4_AgBiBr_8_. Therefore, a clear influence of the specific ammonium spacer cation on the interaction with a given HTM molecule was explicitly shown for both examples CuPc and Pn.

### 3.2 Growth of CuPc or Pn on 2D-Modified 3D Cs_2_AgBiBr_6_

#### 3.2.1 Effects of 2D-Modification

Before analyzing the growth mechanisms of the organic hole conductors on the modified surfaces, effects of the surface modification were studied. The morphology of an as-prepared pristine Cs_2_AgBiBr_6_ film is shown as an AFM height image in Figure 6(a). The modification with 0.05 mol/L BABr in isopropanol leading to samples, which in the following are denoted as “BABr-modified”, showed the formation of some additional small grains and of some large terrace-like grains, as can be seen in Figure 6(b). The modification seemed to affect the whole surface. The small grains showed heights of 2–4 nm on top of the underlying Cs_2_AgBiBr_6_ grains. This height can serve as the best estimate of the thickness of the 2D-modified layer. Grazing incidence XRD (GI-XRD), depicted in Figure 6(e), revealed the presence of quasi-2D perovskite phases. Bilayer BA_2_CsAgBiBr_7_ (*n* = 2)^25^ was identified by a peak at 2θ = 4.5° belonging to the (001)-plane with an interplanar spacing of 1.96 nm, fitting to the layer thickness estimated from the observed height (2–4 nm) of the small grains which would then represent 1–2 repeating units of BA_2_CsAgBiBr_7_ in the [001]-direction. Further, a phase of presumably higher n was found, characterized by a signal at 2θ = 8.7°. The underlying Cs_2_AgBiBr_6_ phase showed the reflections expected from literature,^74^ which were also found following 2D-modification. The observed effects on the morphology were consistent with the literature,^13^ where additional small grains on top of the double perovskite layer were also found and assigned to the bilayer phase. There, it was also pointed out that only the top of the Cs_2_AgBiBr_6_ layer was converted to the low-dimensional phases, since no significant change in the total thickness of the perovskite layer was found.^13^ The work function was slightly decreased by BABr-modification (Figures S7 and S8, (a) compared to (g), respectively).

**Figure 6:**
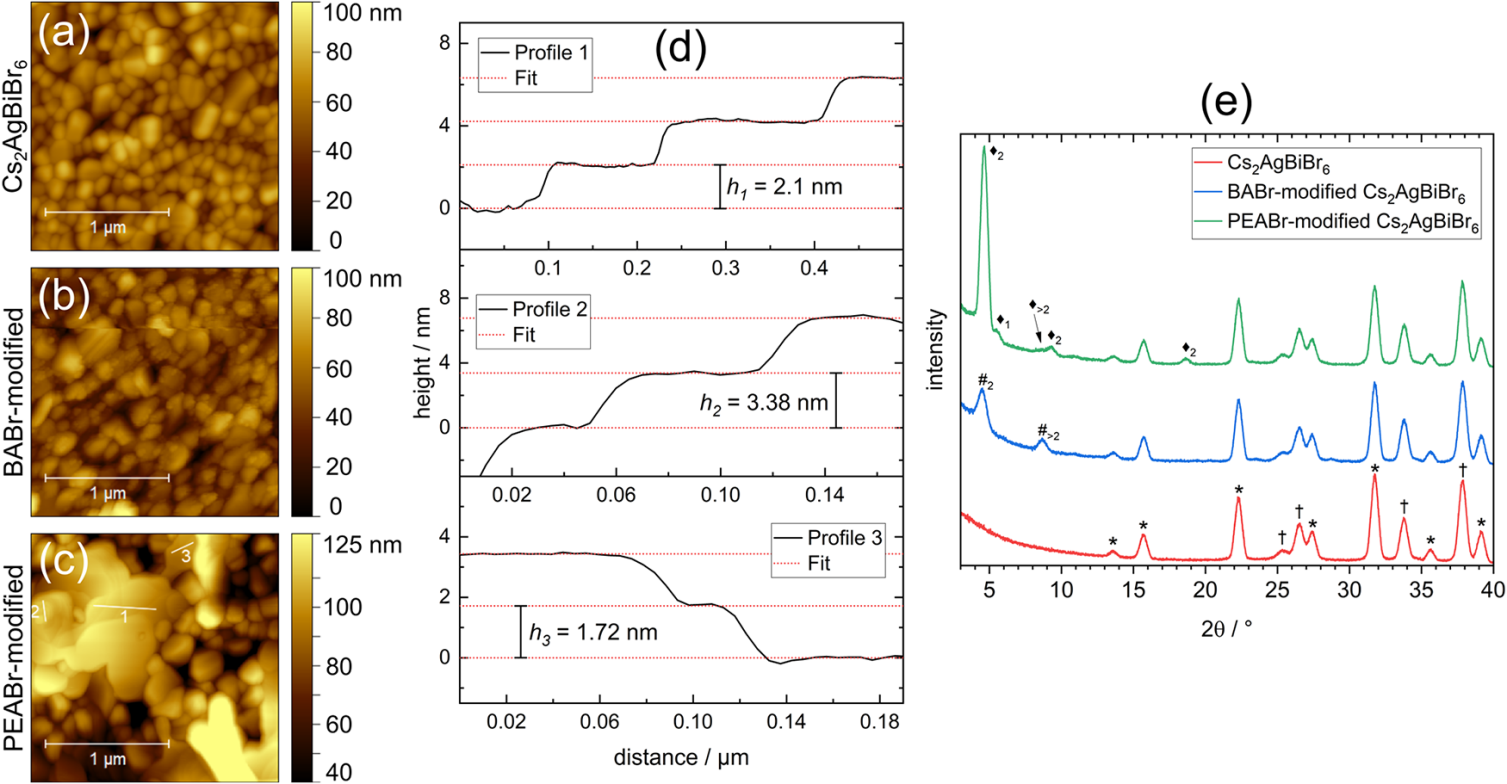
AFM height images of a 135 nm thick pristine (a), BABr-modified (b) and PEABr-modified (c) Cs_2_AgBiBr_6_ thin film. The false color range in (c) was chosen to show step edges as clearly as possible. (d) Step-height analysis of profiles marked in (c). (e) GI-XRD measurements of thin films shown in (a)–(c). Signals arising from Cs_2_AgBiBr_6_ are marked by (*), from the substrate (TiO_2_ on FTO) by (†), from BA-layered phases by (#_n_), and from PEA-layered phases by (⧫_n_). The subscript n indicates the number of octahedral layers between the spacer cations of the respective phase.

In contrast, the surface modification with 0.05 mol/L PEABr in isopropanol, denoted as “PEABr-modified” and shown in Figure 6(c), resulted in the formation of extended flat layered structures with well-defined terraces. The Cs_2_AgBiBr_6_ surface was converted about half-way, with the other half of obviously unchanged grains of Cs_2_AgBiBr_6_ clearly preserved. The large terraces smoothed the surface by replacing numerous 3D perovskite grains but preserved the overall waviness. Among these two regions we detected a large difference in work function (Figures S7­(m) and S8­(m)). In comparison to the 3D perovskite grains with a value of 4.2–4.4 eV, just slightly lower compared to pristine Cs_2_AgBiBr_6_ (4.5 eV), the work function of the 2D islands was significantly lowered to 3.7–3.8 eV if oriented parallel to the substrate (Figures S7 and S8, (a) compared to (m), respectively). Several phases were identified by XRD (Figure 6(e)). The most intense signal at 2θ = 4.6° with higher orders at 9.3° and 18.6° is assigned to PEA_2_CsAgBiBr_7_ ^14^ (*n* = 2), whereas the signals at 2θ = 5.5° caused by PEA_4_AgBiBr_8_ ^26^ (*n* = 1), and at 2θ = 8.7°, indicative of a phase with larger *n*, appeared very weak. The same phases were also found in previous studies, with, however, clear dominance of the *n* = 1 phase.^14^ In Figure 6(d), we show height profiles of the PEABr-modified film exhibiting steps whose height matched well with the deflection angles seen in XRD. The height difference of 2.1 nm between terraces in profile 1 matched with the phase of larger n, whereas those in profiles 2 (3.38 nm) and 3 (1.72 nm) could be assigned to PEA_4_AgBiBr_8_ (*n* = 1), corresponding to 1 or 2 repeating units of the crystal, respectively.^26^ Further, the overall height of the terraced regions above the unchanged 3D perovskite grains was measured to range up to 40 nm while oriented parallel to the surface. Similar thickness has been reported when using fluorinated PEA^+^ ions for modification.^75^ In the more rare cases of nonparallel orientation, edges of the terraces could stick out up to 60 nm from the surface. No closed layer of these terraces was formed. Since the work function, however, was also lowered on the presumably unmodified grains, formation of a thin layer of a 2D phase must be assumed. One could think of very few crystal layers, similar to those seen for the 2–4 nm high grains formed after BABr-modification. The observed changes in work function indicate that PEA^+^ ions are terminating the complete surface.

Although the treatment of 3D Cs_2_AgBiBr_6_ by the two ammonium salts resulted in significantly different sample morphology, the dominant phases consisted of quasi-2D perovskite film surfaces with two octahedral layers between the spacer cations (*n* = 2) for both cations.

#### 3.2.2 Growth of CuPc on 2D-Modified Cs_2_AgBiBr_6_

The morphology of CuPc evaporated onto pristine or 2D-modified Cs_2_AgBiBr_6_ thin films is shown by AFM height images in Figure 7, with corresponding images of the measured work function in Figure S7 in the SI. Starting from pristine Cs_2_AgBiBr_6_ consisting of 50–150 nm sized grains (Figure 7(a)), small CuPc grains of about 25 nm diameter are seen on the considerably larger Cs_2_AgBiBr_6_ grains after the deposition of 2 nm average film thickness (Figure 7(b)). They appeared in clusters in some areas of the sample, leaving other parts uncovered, in line with the overall small amount of CuPc on the surface. Boundaries of the perovskite grains seem to be preferred for CuPc crystallization. At an average thickness of 5 nm, similarly sized CuPc grains covered increasing parts of the perovskite substrate (Figure 7(c)). This trend continued for 10 over 30 to 70 nm average CuPc thickness. The underlying perovskite morphology was more and more covered by CuPc grains of only slightly larger diameter than in the beginning of deposition, and the initial perovskite morphology gradually disappeared (Figure 7(d)–(f)). A growth of CuPc pillars on top of initially formed islands is, thereby, indicated. A very similar morphology was noticed earlier during an analogous experiment of CuPc growth on FASnI_3_.^60^ The corresponding work function (Figure S7), whose distribution is shown in Figure 8(a), started at 4.5 eV for the bare Cs_2_AgBiBr_6_ film and got broader and asymmetric toward lower values upon deposition of CuPc. Masking of the images helped to elucidate the origin of such asymmetry. For this purpose, the work function image was again correlated to the morphology image by transferring the mask to the corresponding height image. This is exemplary shown for 2 nm CuPc on Cs_2_AgBiBr_6_ in Figure S9­(a), which revealed that the agglomerations of small CuPc grains exhibited a higher work function than other parts of the Cs_2_AgBiBr_6_ grains. Since both values were significantly lower than measured for pristine Cs_2_AgBiBr_6_, a monolayer of CuPc seems to have formed either before formation of the round-shaped grains or in parallel, with a lower work function of the monolayer compared to the CuPc grains, presumably caused by different molecular orientation. During further deposition, the work function monotonously decreased with increasing CuPc film thickness, staying asymmetric, consistent with local variations in CuPc thickness. For 50 and 70 nm, the signal turned to a symmetric distribution saturating at around 3.57 eV, speaking for the formation of a homogeneous film. The work function was more than 0.2 eV lower than usually observed on other substrates,^63–65^ possibly due to band bending, as also seen before on MAPbI_3_,^59^ indicating more distinct band bending for Cs_2_AgBiBr_6_ compared to MAPbI_3_. Conversely, an elevated work function compared to literature has been found on FASnI_3_,^60^ indicating band bending in the opposite direction.

**Figure 7:**
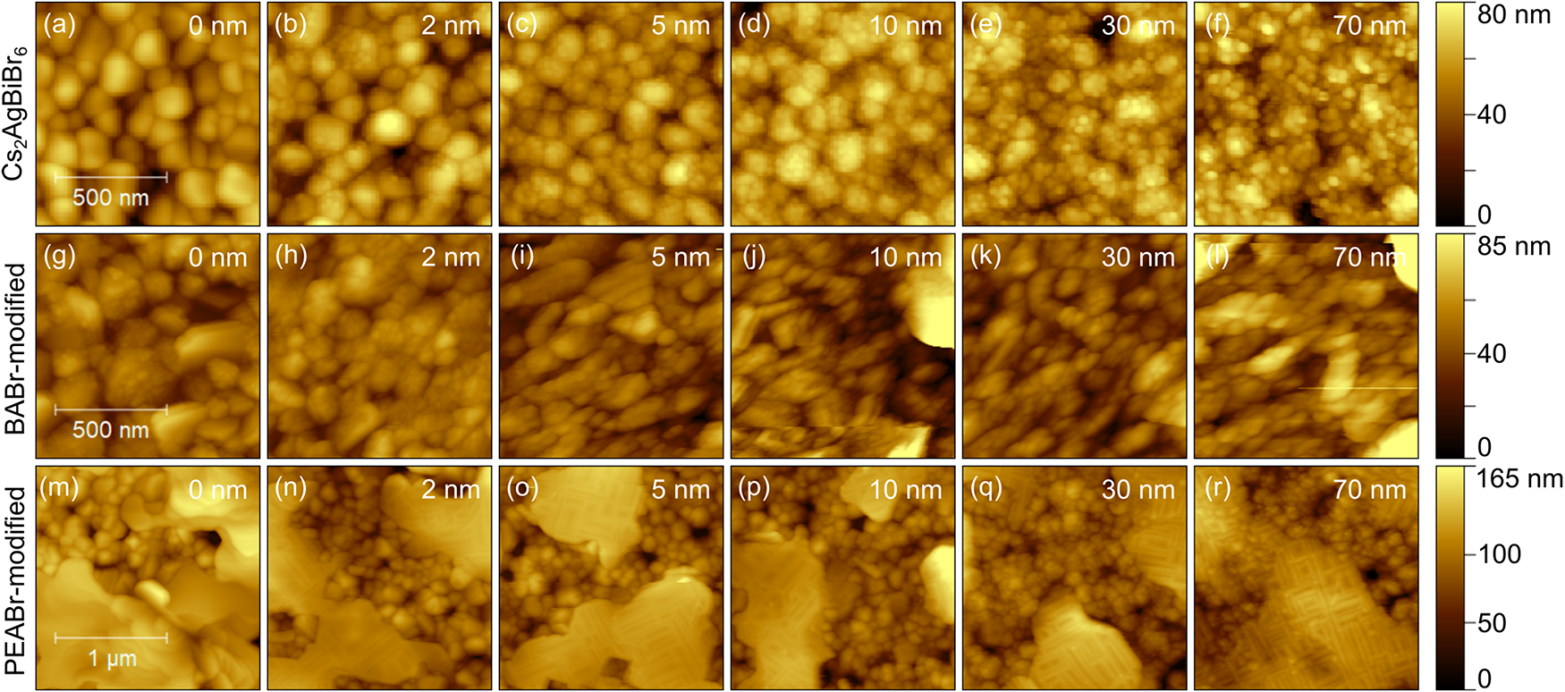
Morphology for different average film thicknesses of CuPc evaporated on top of pristine ((a)–(f)), BABr-modified ((g)-(l)) or PEABr-modified Cs_2_AgBiBr_6_ ((m)–(r)).

**Figure 8:**
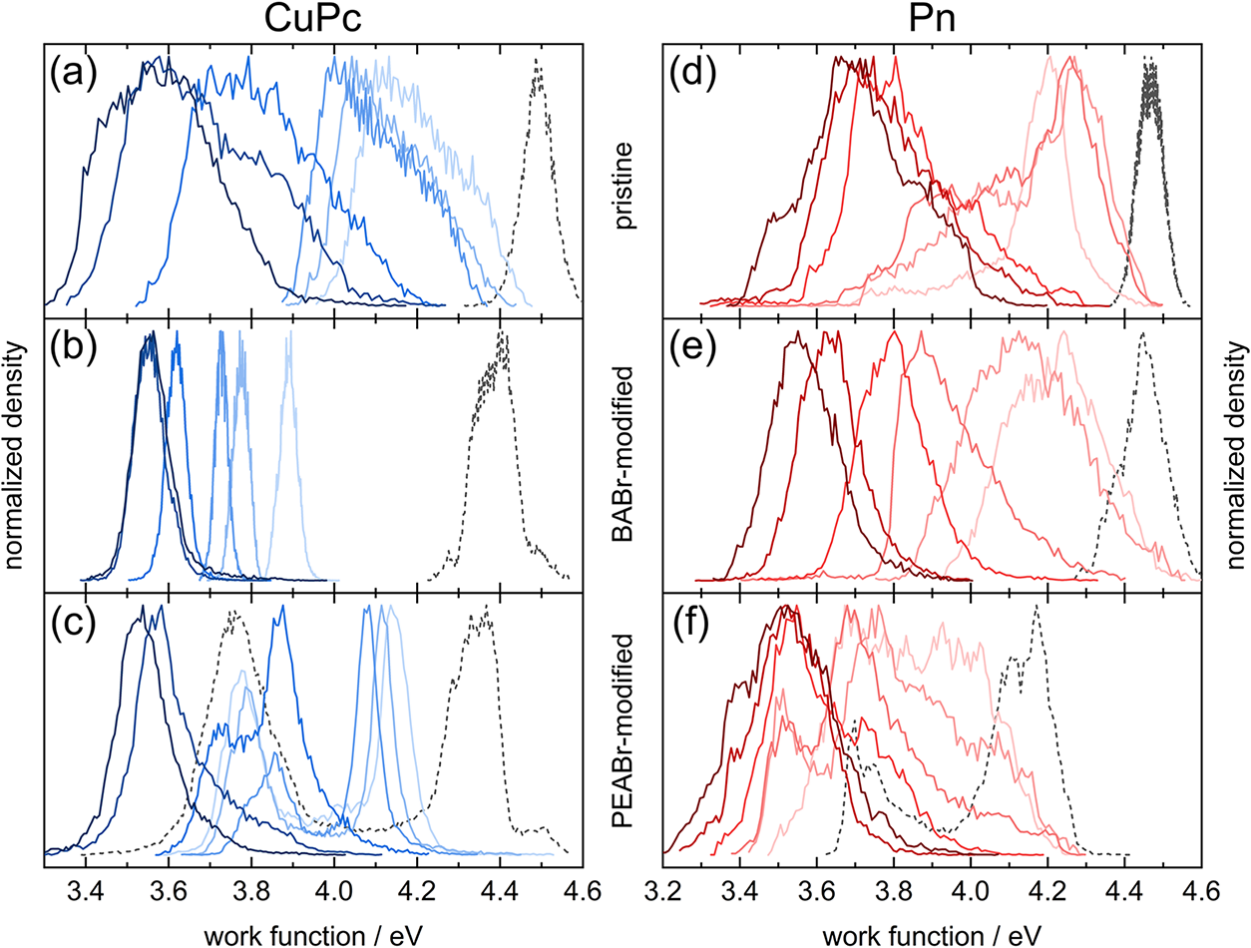
Surface work function distribution of CuPc (blue, (a)–(c)) or Pn (red, (d)–(f)) evaporated on pristine ((a), (d)), BABr-modified ((b), (e)), or PEABr-modified ((c), (f)) Cs_2_AgBiBr_6_ at different average film thicknesses of 2, 5, 10, 30, 50, and 70 nm as illustrated by increasing color intensity. The black dashed profiles represent the respective uncovered perovskite films.

The growth of CuPc on BABr-modified Cs_2_AgBiBr_6_ is depicted in Figure 7(g)–(l). Since many small grains were already present prior to deposition (Figure 7(g)), individual CuPc grains could not be identified after depositing an average film thickness of 2 nm (Figure 7(h)). At this thickness, however, the obtained work function (Figure S7) already showed a significant decrease of about 0.5 eV (Figure 8(b)). Moreover, the distribution was very narrow. This speaks in favor of a very homogeneous coverage by CuPc, indicating Frank-van-der-Merwe growth. With further increasing film thickness, the sample morphology stayed widely constant (Figure 7(i)–(l)), while the work function continuously decreased and saturated at approximately the same value compared to growth on pristine Cs_2_AgBiBr_6_ (Figure 8(a)), but at lower film thickness (Figure 8(b)). Both the constant morphology and the homogeneous work function for each given average film thickness are in good agreement with a layered growth mode. At this point, an improvement of coverage and a much more homogeneous work function can already be identified as a positive effect of the modification with BABr. It seems that surface defects are passivated, and the surface energy is homogenized by the formation of this 2D/3D heterostructure, allowing homogeneous adsorption of a layer of CuPc and its subsequent layer-by-layer growth.

Figure 7(m)–(r) illustrate the growth of CuPc on PEABr-modified Cs_2_AgBiBr_6_, which shows very similar features as observed on pure PEA_4_AgBiBr_8_ as discussed above. On top of the 2D terraces formed by surface modification (Figure 7(m)), CuPc grew well-ordered along well-defined directions. As can be seen from Figure 7(n)–(r), again, needles parallel or perpendicular to each other were formed. In regions of 3D perovskite-like grains, CuPc obviously grew similarly as on pristine Cs_2_AgBiBr_6_ and formed small round-shaped grains on top of the larger perovskite grains (see, e.g., Figure 7(q) vs (m)). These two different growth mechanisms of CuPc were evident for all film thicknesses. The two components of the work function distribution of CuPc on PEABr-modified Cs_2_AgBiBr_6_ (Figure 8(c)) showed quite contrasting changes with increasing film thickness of deposited CuPc. The component arising from growth on 3D grains shifted to lower values, similar to the experiment on pristine Cs_2_AgBiBr_6_, however, with a large shift already at 2 nm and forming a very narrow distribution, more similar to the growth of CuPc on the grains of BABr-modified Cs_2_AgBiBr_6_. Despite the similar morphology of Cs_2_AgBiBr_6_ in the pristine film, the BABr-modified film, and the grainy part of the PEABr-modified film, the distinct values of the work function and their characteristically different shift upon deposition of CuPc show a clear influence of ammonium modification already on these grainy parts of the films. In contrast, the work function component of the 2D terraces of PEA-modified Cs_2_AgBiBr_6_ did not change significantly at low CuPc film thickness. The work function already was quite low; it only started to decrease further at around 30 nm of CuPc. From here on, the work function on the terraces and on the grains was found to be more similar, merged at about 50 nm and saturated at 3.54 eV, in a similar range found for CuPc on pristine or BABr-modified Cs_2_AgBiBr_6_.

The growth of CuPc on the differently modified Cs_2_AgBiBr_6_ films was not only accompanied by different morphologies and differently changing work functions, but also, by significantly different intermolecular coupling (probed by optical absorbance) arising from differences in crystal structure as detected in XRD. On pristine and BABr-modified Cs_2_AgBiBr_6_, mainly the α-phase and a small contribution of the β-phase were found via *in situ* UV–vis transmission measurements, similar as on 2D BA_4_AgBiBr_8_. Only the α-phase was present at low film thickness, while the β-phase appeared at film thicknesses exceeding 20 nm (Figure S12­(c),(d)). The α-phase was also confirmed by XRD (Figure S11­(a)). A significantly more intense XRD signal of CuPc on BABr-modified Cs_2_AgBiBr_6_ emphasized its enhanced crystal order compared to its growth on pristine Cs_2_AgBiBr_6_ (Figure S11­(a)). The structure of CuPc on PEABr-modified Cs_2_AgBiBr_6_ turned out to be almost identical to that on PEA_4_AgBiBr_8_. The α-, β-, and η-phases were present right from the beginning of deposition and grew at a widely constant ratio for moderate film thickness. As on PEA_4_AgBiBr_8_, the relative intensity of the η-signal decreased for CuPc exceeding 20 nm average film thickness (Figure S12­(a),(b)) but was detected in XRD (Figure S11­(a)), indicating a high crystalline order. Therefore, the mere existence of a 2D layered perovskite phase created by PEA^+^ spacer molecules, either in PEA_4_AgBiBr_8_ or on PEABr-modified Cs_2_AgBiBr_6_ induced a nucleation of CuPc also in the η-phase, in addition to nucleation in the more typical α- or β-phases, as exclusively found on pristine or BABr-modified Cs_2_AgBiBr_6_.

#### 3.2.3 Growth of Pn on 2D-Modified Cs_2_AgBiBr_6_

AFM height images during growth of Pn on Cs_2_AgBiBr_6_ are shown in Figure 9 with the corresponding work function images shown in Figure S8. After depositing 2 nm of Pn onto pristine Cs_2_AgBiBr_6_, some grains with a mean diameter of 70 nm were detected (Figure 9(b)), which were also indicated by a contrast in work function (Figure S8­(b)). The work function distribution showed a maximum shifted from almost 4.5 eV before deposition to 4.2 eV for 2 nm Pn and an emerging shoulder at lower energy (Figure 8(d)). Again, masking of the work function image followed by transfer of the mask to the height image (example in Figure S10­(a)), revealed that the shoulder represented Pn grains, whereas the main peak corresponded to the perovskite grains. The shift of the main peak, however, indicates that these areas are covered by Pn molecules. Either growth in a Stranski-Krastanov mode or parallel growth of a Pn layer and crystallization in islands is, thereby, indicated. During further deposition, the Pn grains formed elongated clusters of grains (Figure 9(c),(d)), accompanied by an increase in the shoulder in the work function distribution (Figure 8(d)). Although between 20 and 50 nm full coverage by interconnected Pn grains was reached, the corresponding work function still remained asymmetric, including a shoulder at higher values, while the main feature saturated around 3.7 eV. At 70 nm, a rather uniform grain morphology was obtained (Figure 9(f)). The distribution of the corresponding work function still appeared quite broad, pointing to local variations in Pn thickness, as also directly seen in the sample morphology (Figure 9(f)). The value of 3.7 ± 0.2 eV obtained after saturation is lower than found for Pn on BA_4_AgBiBr_8_ or PEA_4_AgBiBr_8_, or of 3.9–4.1 eV typically reported in literature.^71–73^ This suggests a distinct downward band bending of Pn on Cs_2_AgBiBr_6_. Conversely, for Pn on MAPbI_3_ ^45^ or FASnI_3_,^60^ around 4.2 eV were found, indicating small upward band bending.

**Figure 9:**
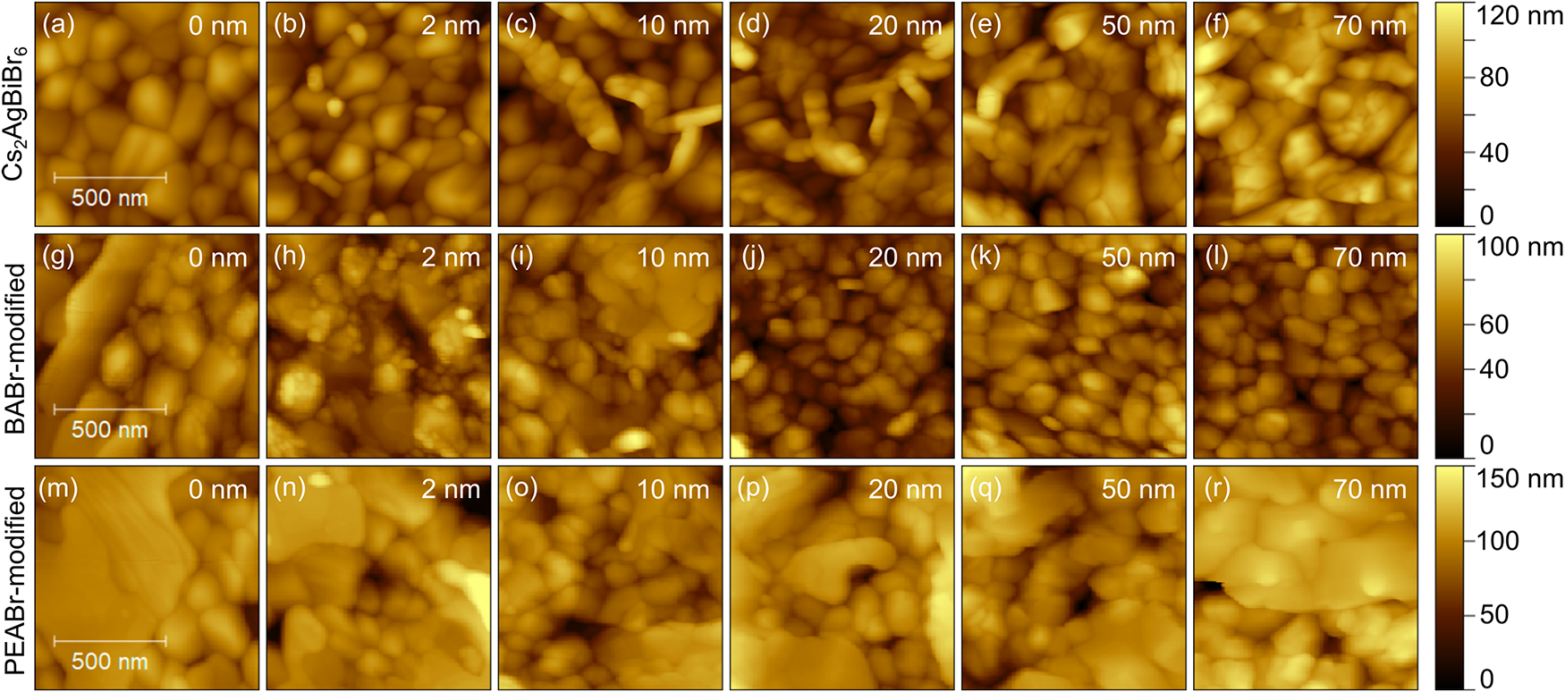
Morphology for different average film thicknesses of Pn evaporated on top of pristine ((a)–(f)), BABr-modified ((g)–(l)), or PEABr-modified Cs_2_AgBiBr_6_ ((m)–(r)).

Figure 9(g)–(l) illustrates the growth of Pn on BABr-modified Cs_2_AgBiBr_6_ with the corresponding work function in Figure S8 (g)–(l). The deposition of an average thickness of 2 nm of Pn resulted in a large number of small grains on the larger perovskite grains (Figure 9(h)). Just based on the height information, it was hard to distinguish Pn grains from perovskite grains since similar features already were present prior to deposition. The corresponding image of the measured work function (Figure S8­(h)), however, helped to identify the Pn grains as distributed spots with a lower work function (Figure S10­(b)). The spots were smaller and more numerous than those on pristine Cs_2_AgBiBr_6_ at a comparable stage of deposition. Due to a more homogeneous coverage with small round Pn grains rather than larger agglomerates, the corresponding distribution of work function (Figure 8(e)) was found at a similar value as on pristine Cs_2_AgBiBr_6_ but exhibited only one main feature. After the deposition of 10 nm Pn, the grain size increased, and Pn almost covered the perovskite film (Figure 9(i)). At this stage, the work function distribution was the broadest and then narrowed again upon further deposition while constantly shifting toward lower values. The morphology did not change further (Figure 9(j)–(l)). At 70 nm Pn on BABr-modified Cs_2_AgBiBr_6_, the peak position of the work function reached 3.55 eV, at the low end of the distribution on pristine Cs_2_AgBiBr_6_ but significantly more confined. This speaks in favor of more homogeneous growth and of a more defined contact alignment of Pn to the perovskite layer, accompanied by a distinct downward band bending.

Figure 9(m)–(r) depicts the height images of the growth of Pn on PEABr-modified Cs_2_AgBiBr_6_, while the corresponding work function images are shown in Figure S8­(m)–(r). After the deposition of 2 nm of Pn, no significant morphological change was detected (Figure 9(n)). The corresponding work function image (Figure S8­(n)), however, showed spots of significantly lowered work function in both layered and grainy regions, respectively, showing that Pn grains had formed throughout the sample. This resulted in a very broad distribution, as seen in Figure 8(f), to which covered and uncovered areas of the perovskite, as well as Pn spots in both regions contributed. Until 20 nm, the signal at higher energy gradually decreased with increasing film thickness, representing the successive coverage of the 3D perovskite grains. A shoulder assigned to Pn grains on the 2D layered regions evolved, which was centered at the later global maximum upon saturation, as emphasized in Figure S10­(c). For the grainy region, saturation was reached at 50 nm, as indicated by a symmetric distribution. At higher film thickness, Pn grew in large terraced islands on top the 2D layered regions similarly as on PEA_4_AgBiBr_8_, albeit at slightly lower thickness, whereas it formed grains on the 3D perovskite regions (Figure 9(q),(r)), which had a similar shape as on pristine and BABr-modified Cs_2_AgBiBr_6_. The work function saturated at 3.52 eV and hence coincided with the value obtained for Pn on BABr-modified Cs_2_AgBiBr_6_. It can be concluded that different growth of Pn was detected on the terraces of the respective 2D layered structure as opposed to regions dominated by 3D perovskite grains: Pn grew in larger dendrite-like islands in the first case, similar as observed on pure 2D double perovskite surfaces of BA_4_AgBiBr_8_ or PEA_4_AgBiBr_8_, or, earlier, on SiO_2_ ^30,43,54–57^ while, in the latter case, Pn formed grains in a similar way as observed on pristine Cs_2_AgBiBr_6_.

X-ray diffractograms of the Pn films (Figure S13­(a)) showed a superposition of the thin film and bulk phases for deposition of Pn on either pristine, BABr-modified or PEABr-modified Cs_2_AgBiBr_6_. Such a finding is well in line with the literature, where a superposition of both phases was often reported, with the proportion depending on thickness and preparation conditions.^30,54,55,58^ A trend toward higher intensity of the Pn signals relative to the respective substrate signals, in particular on the PEABr-modified and to a lesser extent also on the BABr-modified surface, when compared to pristine Cs_2_AgBiBr_6_, speaks in favor of an enhanced crystalline order of Pn on modified Cs_2_AgBiBr_6_.

### 3.3 Device Formation

To check for direct consequences of the discussed modifications in morphology and work function of the deposited HTMs on contact properties, devices in solar cell architecture were exemplary studied for a few test devices in FTO/TiO_2_/Cs_2_AgBiBr_6_ (pristine, BA-, or PEA-modified)/HTM/Au geometry to prove the enhancement of PV activity by a 2D-modification of Cs_2_AgBiBr_6_ for CuPc and Pn as HTMs, as already reported for Spiro-OMeTAD.^13,14^ Since all our HTM films had reached a closed morphology (Figure 7 or Figure 9(f),(l),(r), respectively) and a homogeneous work function (Figure 8) for an HTM thickness of 70 nm, this thickness was chosen for the test devices. Reliably working devices were obtained and the photovoltaic parameters of the different device configurations are shown in Table 1. The current–voltage characteristics of all devices confirmed the typical character of a photovoltaic junction, as shown in Figure 10 for the device using PEABr-modified Cs_2_AgBiBr_6_ and CuPc as HTM, which performed best in our work. The performance of this device did not quite reach that of related cells in literature but reached the same open-circuit potential (Table 1).^14^ The smaller current that was presently obtained can easily arise from an additional mesoporous-TiO_2_ layer used in ref 14, use of doped Spiro-OMeTAD^14^ as an optimized HTM as opposed to typically quite poorly conducting CuPc, and other subtle but decisive differences in device architecture. Comparing the different devices prepared in this work, the PEABr-modification effectively improved the open-circuit potential for both CuPc and Pn as HTM. The short-circuit current density is enhanced as well, in particular in the case of CuPc. Although the devices with BABr-modification showed similar fill factors, their performance lags behind that of the unmodified devices. Nevertheless, a positive effect on the stability over time was confirmed for both 2D-modifications (Figure S15). Independent of the HTM, the BABr-modification improved the stability of the short-circuit current density, while the PEABr-modification, in addition, improved the stability of the open-circuit potential. This speaks in favor of enhanced device performance. Therefore, the positive role of a 2D-modification of Cs_2_AgBiBr_6_ for device enhancement was also shown for the simpler device architecture and HTMs CuPc and Pn investigated in this work, for which a clear optimization of contact formation to Cs_2_AgBiBr_6_ was independently shown in the morphology and work function of films during contact formation.

**Figure 10:**
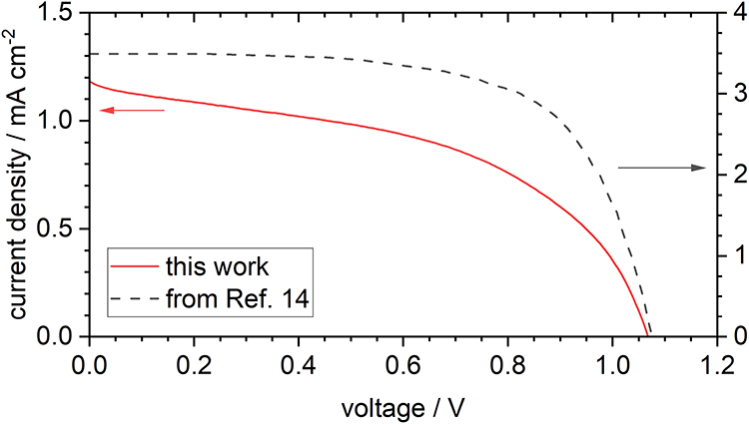
Current–voltage characteristics of an FTO/TiO_2_/PEABr-modified Cs_2_AgBiBr_6_/CuPc/Au device (red solid line) compared to a comparable device (FTO/TiO_2_/mesoporous-TiO_2_/PEABr-mod. Cs_2_AgBiBr_6_/Spiro-OMeTAD/Au) from literature^14^ (gray dashed line).

**Table 1:** Photovoltaic Parameters of Devices Prepared in This Work and Comparison to a Comparable Device in Literature

configuration	*U*_OC_/V	*j*_SC_/mA cm^–2^	FF	PCE/%
FTO/TiO_2_/Cs_2_AgBiBr_6_/CuPc/Au	0.85	0.69	0.31	0.18
FTO/TiO_2_/BABr-mod. Cs_2_AgBiBr_6_/CuPc/Au	0.42	0.14	0.33	0.02
FTO/TiO_2_/PEABr-mod. Cs_2_AgBiBr_6_/CuPc/Au	1.07	1.18	0.49	0.61
FTO/TiO_2_/Cs_2_AgBiBr_6_/Pn/Au	0.73	0.40	0.34	0.10
FTO/TiO_2_/BABr-mod. Cs_2_AgBiBr_6_/Pn/Au	0.48	0.13	0.33	0.02
FTO/TiO_2_/PEABr-mod. Cs_2_AgBiBr_6_/Pn/Au	0.99	0.44	0.36	0.16
FTO/TiO_2_/mesoporous-TiO_2_/PEABr-mod. Cs_2_AgBiBr_6_/Spiro-OMeTAD/Au (ref 14)	1.07	3.50	0.66	2.47

### 3.4 Comparison of Substrates and HTMs

To reveal the origin of the reported improvements of solar cell performance^13,14^ by treatment of Cs_2_AgBiBr_6_ with organic ammonium and, in particular, PEA^+^ or BA^+^ salts before application of an HTM, a comparison to their growth on the BA_4_AgBiBr_8_ PEA_4_AgBiBr_8_ model substrates is carried out for both hole transport materials. The observed morphologies and crystal phases of CuPc or Pn as HTMs are summarized in Table 2. On pristine Cs_2_AgBiBr_6_, both CuPc and Pn preferentially nucleated in large grain agglomerations. Complete and homogeneous coverage was achieved at a high film thickness. Such agglomerations were not seen on either BABr- or PEABr-modified Cs_2_AgBiBr_6_, nor on the BA_4_AgBiBr_8_ or PEA_4_AgBiBr_8_ 2D double perovskite model substrates. BABr-modification resulted in a very homogeneous coverage by the HTM molecules even in the initial stages of deposition, in line with the observed film growth very similar to that on BA_4_AgBiBr_8_ or other common substrates, in particular for Pn. The ordered growth of short CuPc needles seen on BA_4_AgBiBr_8_ at low film thickness could not be observed on BABr-modified Cs_2_AgBiBr_6_ since the modification mostly preserved the morphology of the underlying 3D phase rather than forming layered terraces. On the other hand, the PEABr-modification led to layered regions aside from the typical 3D perovskite grains and, thereby, allowed a significantly more homogeneous growth of the HTMs. Long CuPc needles or layered dendritic Pn islands were found on the layered terraces formed following the PEABr-modification. Especially, the results obtained on the layered regions show clear analogy to the observations on PEA_4_AgBiBr_8_, as CuPc and Pn both grew similarly on either of the two, although they had different work functions. Hence, the general influence of such terraced double perovskite surfaces on the growth of the HTMs was indicated. Rather than forming homogeneously distributed round grains, the 2D layered terraces induced epitaxial growth of well-ordered CuPc needles or large, layered dendritic Pn islands with well-defined layer steps in accordance with the crystal structure, respectively. A deviation of the ideal planar spacing of Pn for the very first layer of molecules on BA_4_AgBiBr_8_, which was not seen on PEA_4_AgBiBr_8_, however, demonstrated a different interaction of the Pn molecules with the terraces containing either BA^+^ or PEA^+^ spacer cations. While no significant variation of crystal phases was identified for Pn, the observed differences in morphology for CuPc were accompanied by different crystal phases in the film (Table 2). On pristine and BABr-modified Cs_2_AgBiBr_6_, CuPc nucleated in the α-phase, and the short CuPc needles on BA_4_AgBiBr_8_, were in the α-phase as well. In contrast, XRD and UV–vis measurements revealed that the long CuPc needles found at low film thickness on PEA_4_AgBiBr_8_ and on the terraced areas formed following the PEABr-modification of Cs_2_AgBiBr_6_ nucleated in the η-phase. For thicker films, these needles were of medium length only and an increasing amount of the α-phase was seen. From these observations, it can be presumed that CuPc forms needles on layered double perovskite terraces, but only terraces based on PEA^+^ spacer ions allow nucleation in the η-phase, forming the observed long needles. As a result, a significant influence of such terraces compared to a polycrystalline grain structure was observed on the growth of both studied HTMs, and it can reasonably be assumed that such an influence extends toward other HTM materials.

**Table 2:** Schematic Summary of the Observed Film Morphologies and Crystal Phases of CuPc and Pn on the Different Double Perovskite Substrates

substrate	CuPc < 50 nm	CuPc > 50 nm	Pn
Cs_2_AgBiBr_6_	round grains in α-phase	round grains in α-phase	round grains in thin film and bulk phase
BABr-mod. Cs_2_AgBiBr_6_			
BA_4_AgBiBr_8_	short needles in α-phase	short needles in α-phase	dendritic islands in thin film phase
PEA_4_AgBiBr_8_	long needles in η-phase	needles of medium length in α- and η-phases	
PEABr-mod. Cs_2_AgBiBr_6_			dendritic islands in thin film and bulk phase

Figure 11 summarizes the measured work functions of different Cs_2_AgBiBr_6_-based samples (3D, BABr-mod., PEABr-mod.) and changes observed upon deposition of either HTM toward full coverage. Following PEABr-modification, the work function on 3D perovskite grains was found almost identical to that following modification by BABr, with both just slightly lower than that of pristine Cs_2_AgBiBr_6_, all showing a similar morphology. On the 2D layered terraced regions that were formed by PEABr-modification, however, the work function was significantly decreased, already close to the values of CuPc and Pn (Figure 11). Nevertheless, the work function of the deposited HTMs was affected by the respective spacer cation, as most clearly seen for the phase-pure 2D samples. On BA_4_AgBiBr_8_, both HTMs featured work functions comparable to their values typically reported in literature. The same held for Pn on PEA_4_AgBiBr_8_, whereas a significant lowering occurred for CuPc on PEA_4_AgBiBr_8_, accompanied by a different crystal structure and therefore a different molecular arrangement. Following 2D-modification of Cs_2_AgBiBr_6_ by either BABr or PEABr, the work function of the deposited HTMs was found to be significantly more defined than that on Cs_2_AgBiBr_6_ (Figure 8), while its value was mostly maintained, aside from a slightly lower value for Pn. Consequently, the hole transfer from Cs_2_AgBiBr_6_ to the HTMs is facilitated after 2D-modification by a split into two consecutive steps of smaller energy difference via an additional intermediate energy level as opposed to one big step needed in the unmodified interface (Figure 11). Facile transition to this intermediate layer can further serve to suppress hole recombination in the absorber layer. Despite different effects on film morphology, such contact improvement of the HTMs to the perovskite layers was achieved by both BA^+^ and PEA^+^ in a similar way. A positive effect of these changes in contact formation was directly confirmed in complete model solar cells with CuPc or Pn serving as HTMs. The effect reported earlier for cells using established hole conductors and cell architectures,^13,14^ could, thereby, be directly traced back to differences in contact formation in the present model experiments performed during contact formation.

**Figure 11:**
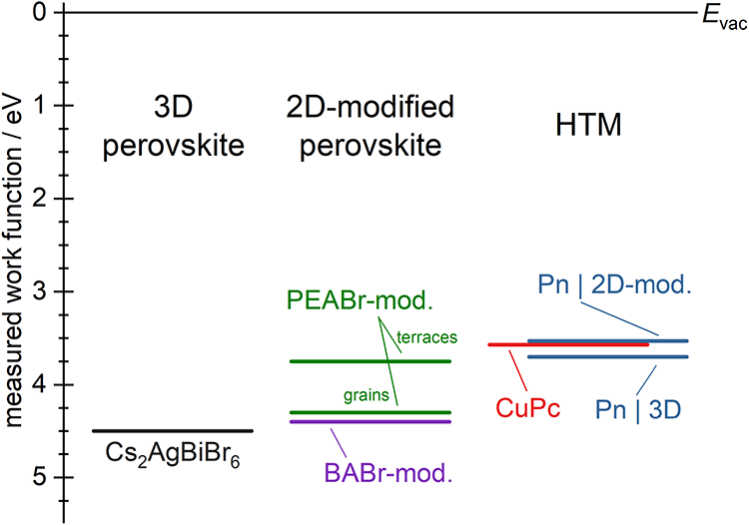
Summary of the measured work function for Cs_2_AgBiBr_6_ in either the pristine 3D structure or following 2D-modification by either BABr or PEABr and following its coverage by CuPc or Pn as model HTM, respectively. Upon PEABr-modification, significantly different values were found on the observed grains or terraces, respectively. As opposed to CuPc, which showed roughly the same work function for complete coverage of all substrates, Pn showed two different values on either pristine or 2D-modified Cs_2_AgBiBr_6_.

## 4 Conclusions

In this work, we directly showed how the interaction of spacer cations BA^+^ or PEA^+^ with HTM molecules can be used to tune the growth mechanism as well as the contact formation of the latter on Cs_2_AgBiBr_6_. The spacer cations were used either in phase-pure 2D double perovskite films of BA_4_AgBiBr_8_ or PEA_4_AgBiBr_8_ or in appropriate 2D interphases on 3D Cs_2_AgBiBr_6_. Both BABr- and PEABr-modifications of Cs_2_AgBiBr_6_ produced substrates, on which the model HTMs CuPc and Pn grew significantly more homogeneously than on pristine Cs_2_AgBiBr_6_. The strongly contrasting growth modes of the HTMs on either the obtained 2D-perovskite terraces or Cs_2_AgBiBr_6_-like grains with significantly differing work functions provided significantly different conditions for charge transfer. Such differences explain the reported improvement of perovskite solar cells by 2D-modification prior to HTM deposition.^13,14^ Such an improvement was confirmed for model devices directly employing the 2D interphases and vapor-deposited HTMs prepared in this work. Local differences found on given BABr- or PEABr-modified Cs_2_AgBiBr_6_ samples can lead to a local variation of contact formation. Such a possibility should be investigated further to gain further improvement of perovskite solar cells. Most clearly observed at low coverages of the HTMs, BABr- or PEABr-modification of Cs_2_AgBiBr_6_ led to considerably more homogeneous film growth. Such modifications should, therefore, allow reduction of the HTM film thickness in devices and, hence, decrease their series resistance. The observed consequences of 2D-modification of Cs_2_AgBiBr_6_ by BABr or PEABr were analyzed in detail by comparison to HTM growth on phase-pure 2D BA_4_AgBiBr_8_ or PEA_4_AgBiBr_8_ model surfaces. The key aspects found in this work, namely, an improved homogeneity of film growth and a more homogeneous alignment of energy levels, have the potential to further extend the already known advantages of 2D/3D heterostructures in future perovskite solar cells.

## Supplementary Material



## Notes

Additional KPFM height and work function images, detailed height profiles, illustration of masking used for correlation of morphology and work function, XRD and *in situ* UV–vis analysis of CuPc and Pn thin films, XRD and confocal laser microscopy images to discuss solvent engineering of PEA_4_AgBiBr_8_, and stability of photovoltaic parameters in test devices (PDF)

## Abbreviations

AFM: atomic force microscopy
BA: butylammonium
CuPc: copper phthalocyanine
HTM: hole transport material
KPFM: Kelvin probe force microscopy
PEA: phenethylammonium
Pn: pentacene
XRD: X-ray diffraction
