# Supplementary material for: Effect of a 2D-Modification of Cs2AgBiBr6 on Nucleation and Contact Formation of Subsequently Deposited Hole Transport Layers as Revealed by In Situ Growth Studies
Source: ACS Appl Mater Interfaces. 2026 Jan 30;18(5):9248–62. doi: 10.1021/acsami.5c24299 (PMC12903113; doi:10.1021/acsami.5c24299)
Supplement: Supplementary file 1 [file am5c24299_si_001.pdf]

Supporting Information

Effect of a 2D-Modification of $Cs_2AgBiBr_6$ on Nucleation and Contact Formation of Subsequently Deposited Hole Transport Layers as Revealed by *in-situ* Growth Studies

Tim P. Schneider[a,b], *Fabian Schmitz*[b], *Teresa Gatti*[b,c] *and Derck Schlettwein*[a,b,*]

[a] Institute of Applied Physics, Justus Liebig University Giessen, Heinrich-Buff-Ring 16, 35392 Gießen, Germany

[b] Center of Materials Research (ZfM), Justus Liebig University Giessen, Heinrich-Buff-Ring 16, 35392 Gießen, Germany

[c] Department of Applied Science and Technology, Politecnico di Torino, Corso Duca degli Abruzzi 24, 10129 Torino, Italy

* corresponding author at email: schlettwein@uni-giessen.de

1. Additional aspects regarding morphology and work function during deposition of HTMs

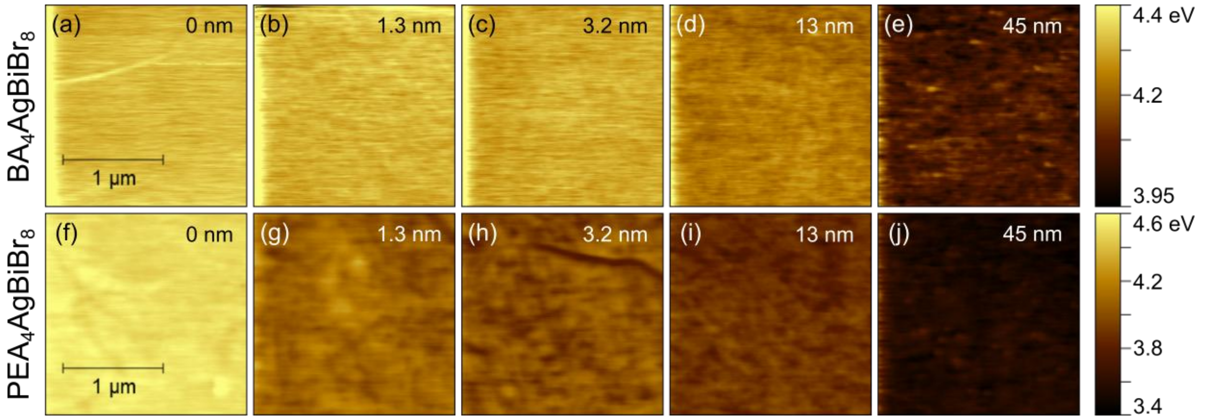

Fig. S1. Work function for different average film thickness of CuPc evaporated on top of $BA_4AgBiBr_8$ ((a)-(e)) or $PEA_4AgBiBr_8$ ((f)-(j)).

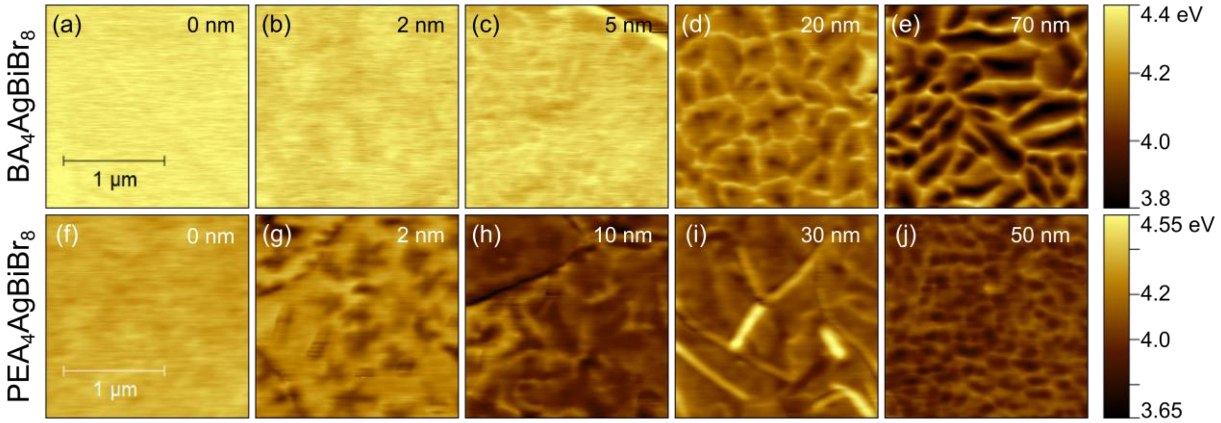

Fig. S2. Work function for different average film thickness of Pn evaporated on top of $BA_4AgBiBr_8$ ((a)-(e)) or $PEA_4AgBiBr_8$ ((f)-(j)).

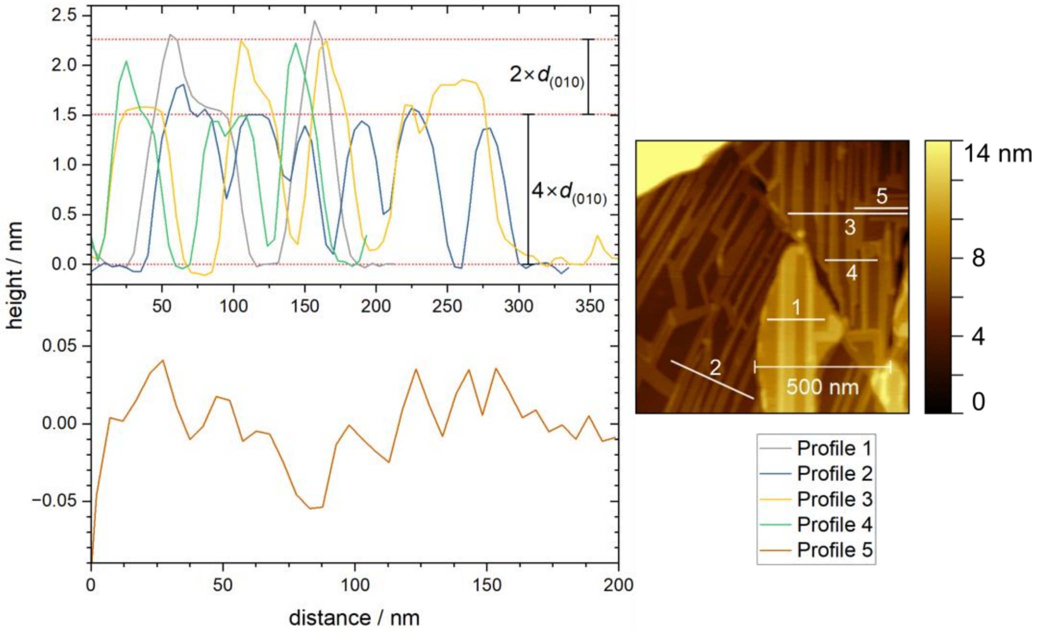

Fig. S3. AFM height image of 0.63 nm CuPc on $PEA_4AgBiBr_8$ (right) with height profiles perpendicular (1-4) and along (5) the observed needles and analysis of extracted profiles (left). Multiples of the (010)-lattice constant of the CuPc η-phase (0.375 nm),[1] are shown for comparison.

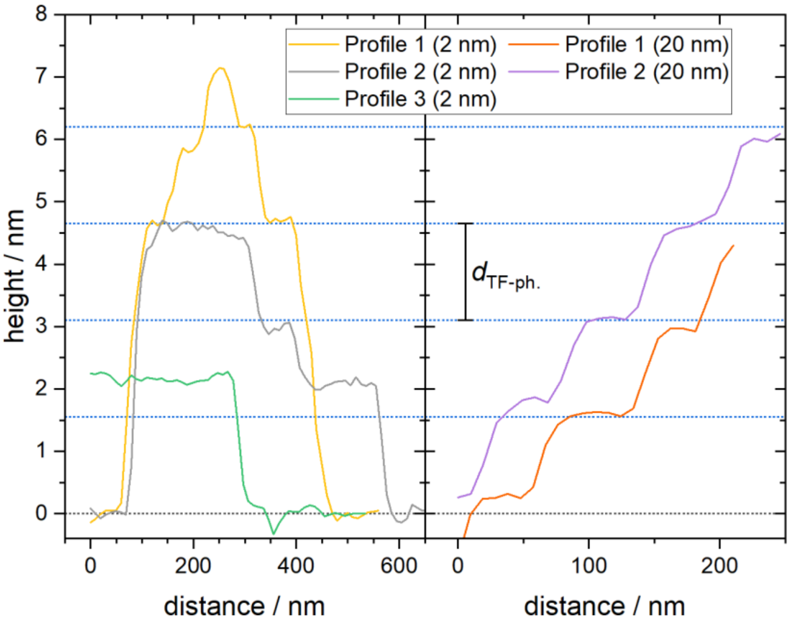

Fig. S4. Height profiles extracted from Fig. 4 (b) (left) and (d) (right) of the main text. The interplanar spacing of the Pn thin film phase is marked as $d_{TF\text{-}ph.}$ (1.55 nm)[2–4] since only this phase was confirmed by XRD.

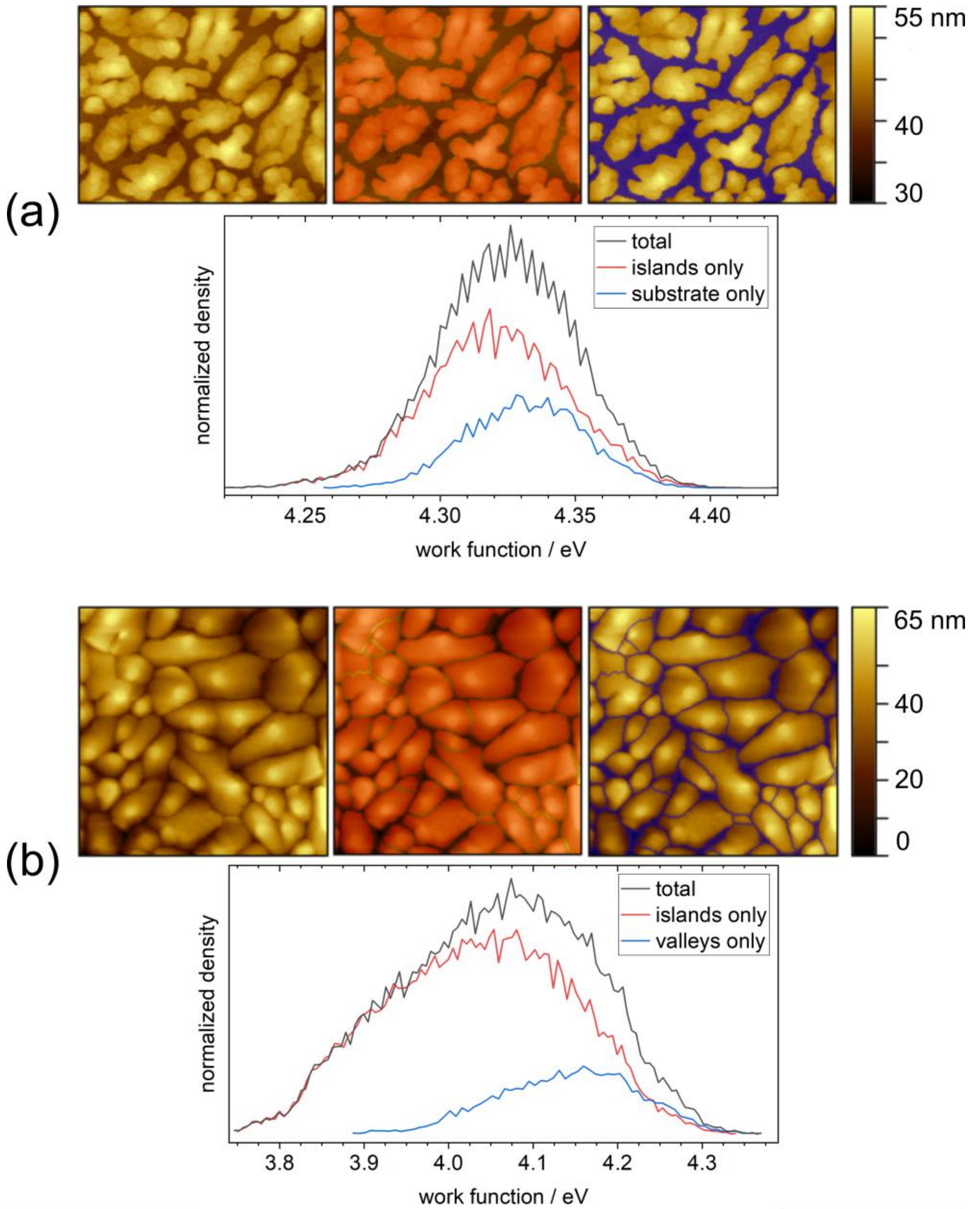

Fig. S5. Illustration of masking used for correlation of morphology (Fig. 4 (c) and (e) of the main text) and work function (Fig. S2 (c) and (e)) for the growth of Pn on $BA_4AgBiBr_8$, exemplary shown for two stages (5 nm (a) and 70 nm (b)). In the AFM height image (left), Pn islands are marked in red (middle), and the substrate or Pn valleys are marked in blue (right). The distribution of work functions, extracted by transferring the respective masks to the corresponding work function images, is shown below.

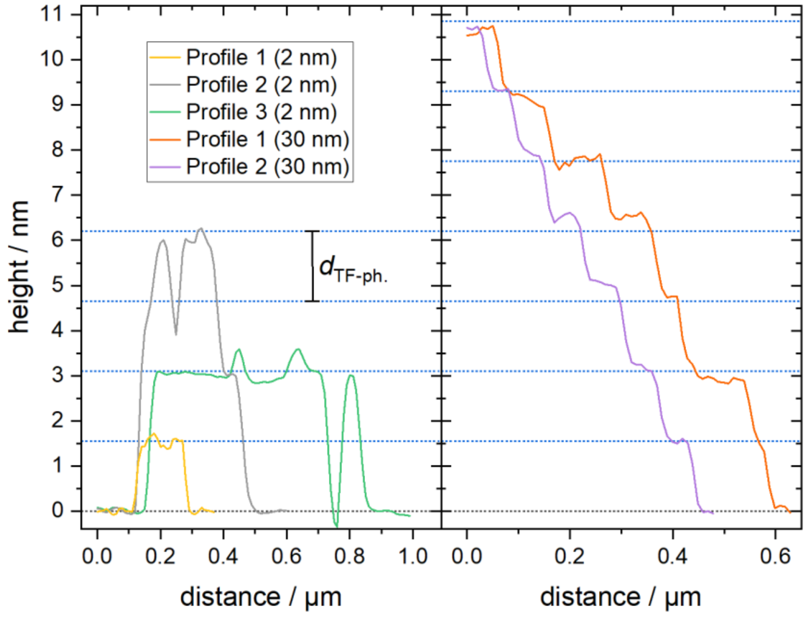

Fig. S6. As in Fig. S4 but for height profiles extracted from Fig. 4 (g) (left) and (i) (right) of the main text.

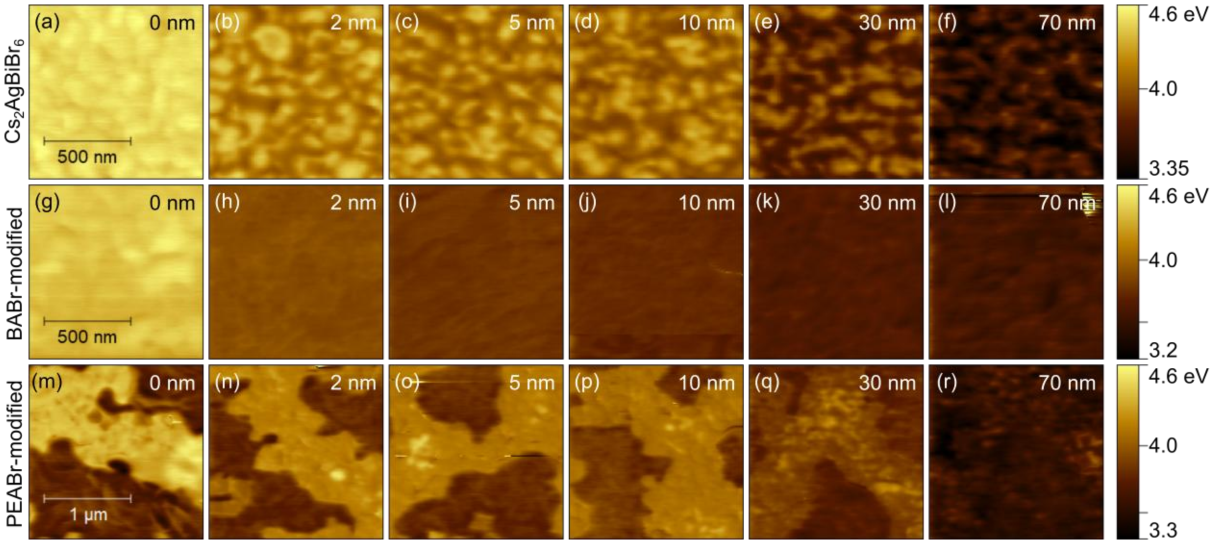

Fig. S7. Work function for different average film thickness of CuPc evaporated on top of pristine ((a)-(f)), BABr-modified ((g)-(l)) or PEABr-modified $Cs_2AgBiBr_6$ ((m)-(r)). Histograms of the work function in the respective measurements are depicted in Fig. 8 (a)-(c) of the main text.

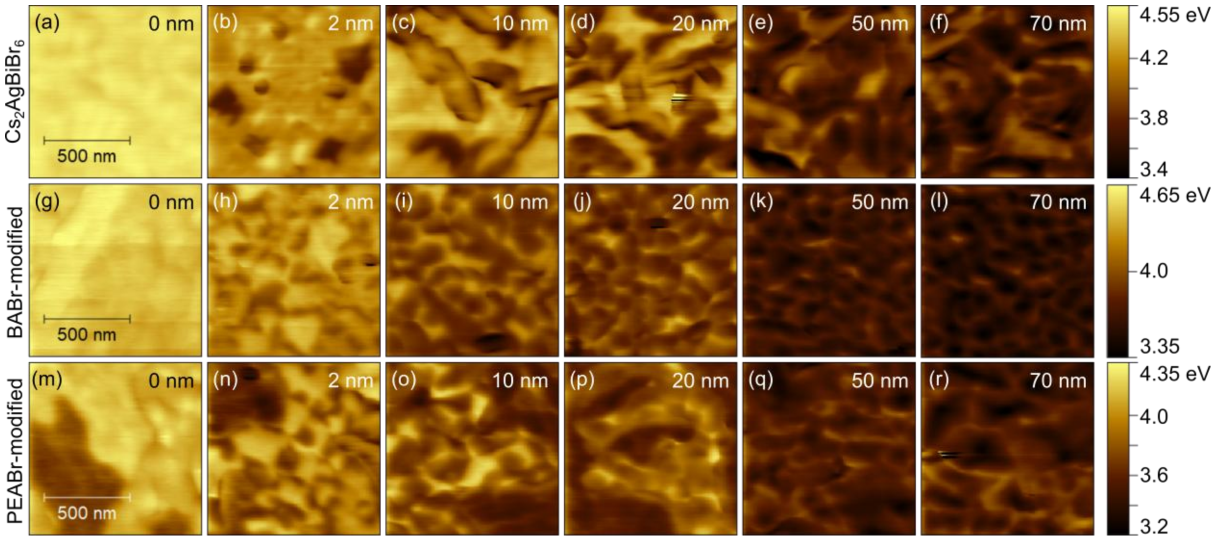

Fig. S8. Work function for different average film thickness of Pn evaporated on top of pristine ((a)-(f)), BABr-modified ((g)-(l)) or PEABr-modified $Cs_2AgBiBr_6$ ((m)-(r)). Histograms of the work function in the respective measurements are depicted in Fig. 8 (d)-(f) of the main text.

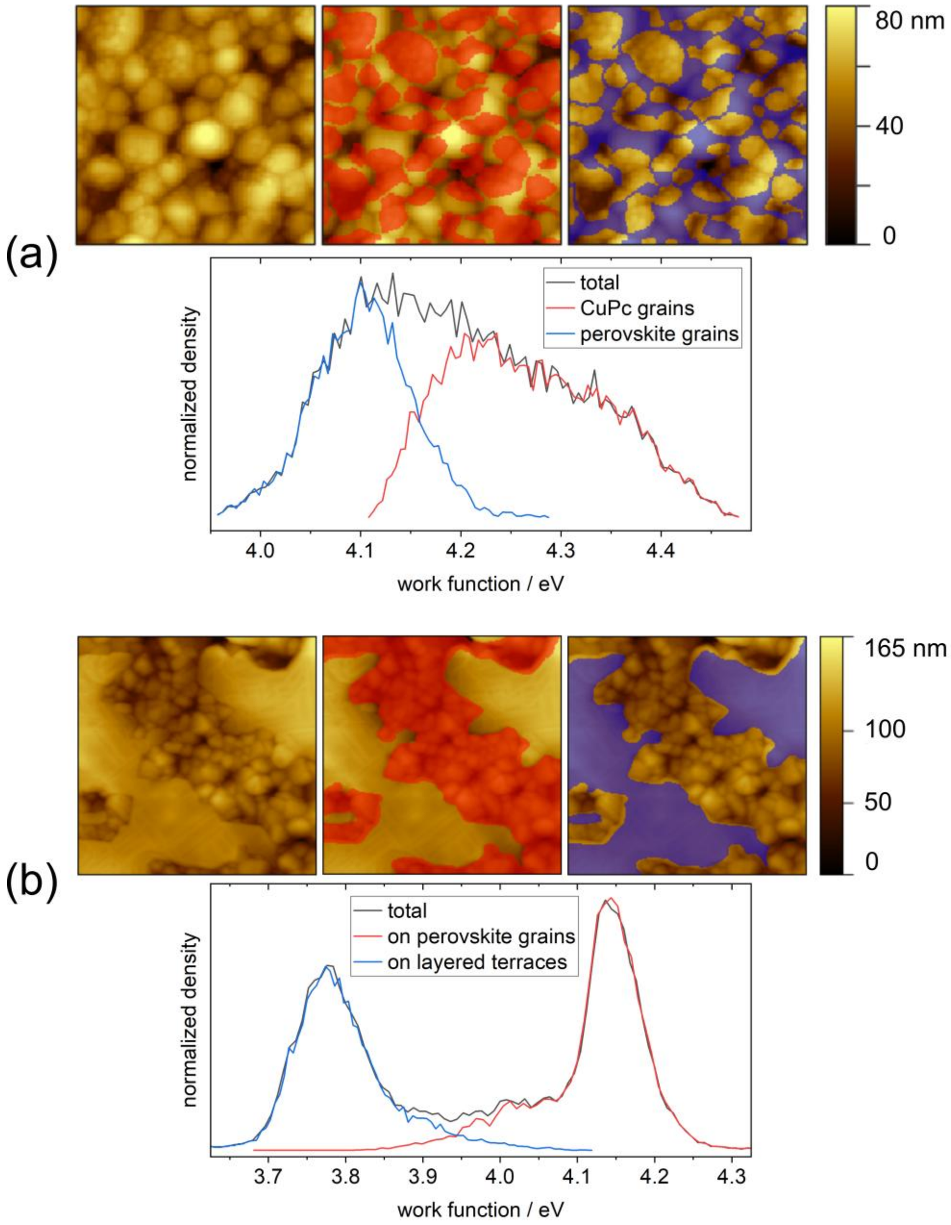

Fig. S9. Illustration of masking used for correlation of morphology (Fig. 7 (b) and (n) of the main text) and work function (Fig. S7 (b) and (n)) for the growth of CuPc on pristine ((a), 2 nm) and PEABr-modified $Cs_2AgBiBr_6$ ((b), 2 nm), respectively. Masks were applied to the work function images, the work function of the differently masked areas was plotted into the measured distribution of work functions (see also Fig. 8 of the main text) and the areas were identified by transferring the respective masks to the corresponding morphology images.

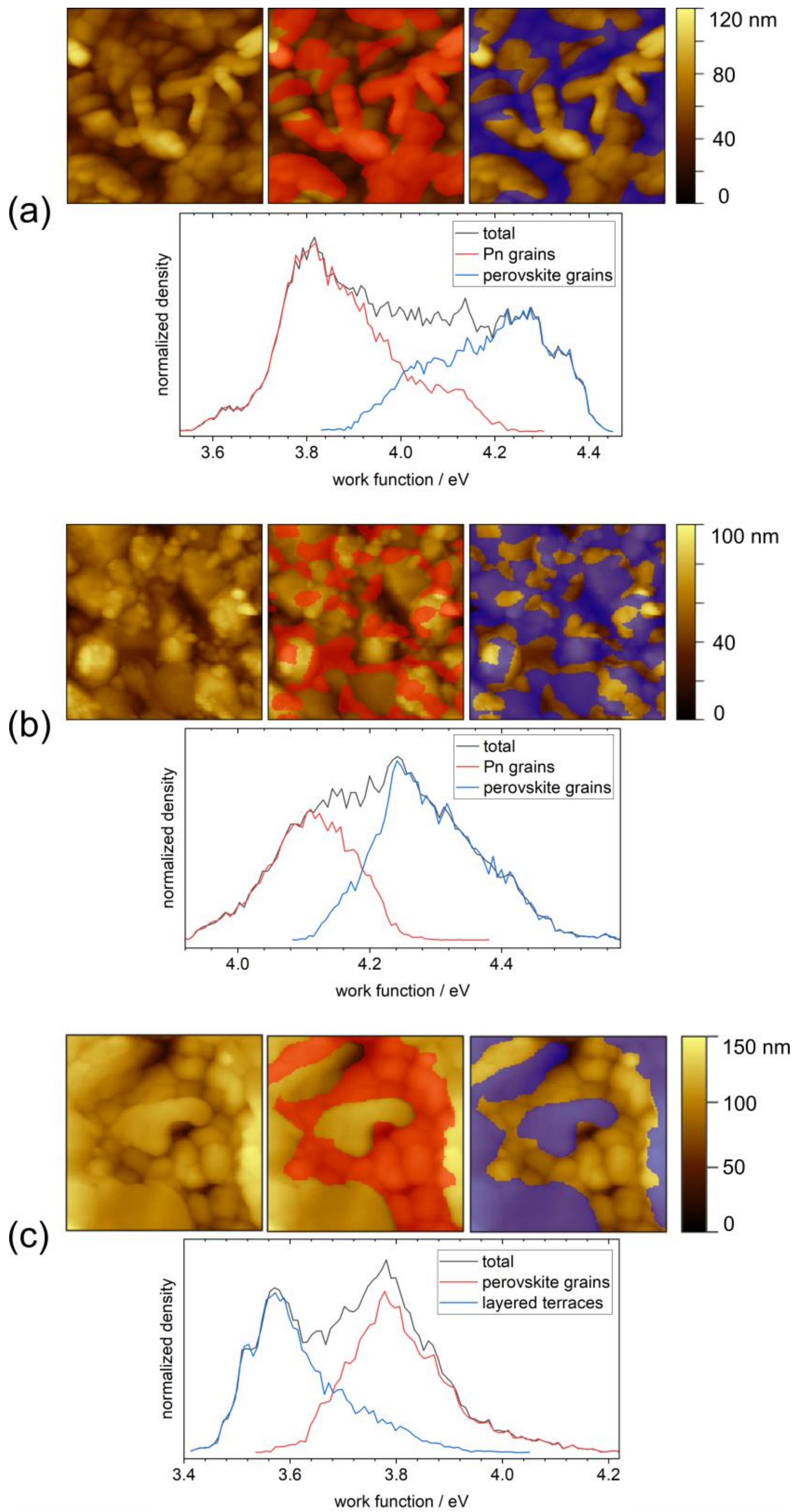

Fig. S10. Illustration of masking used for correlation of morphology (Fig. 9 (d), (h) and (p)) and work function (Fig. S8 (d), (h) and (p)) for the growth of Pn on pristine ((a), 20 nm), BABr- ((b), 2 nm) and PEABr-modified ((c), 20 nm) $Cs_2AgBiBr_6$, respectively. Masks were applied to the work function images, the work function of the differently masked areas was plotted into the measured distribution of work functions (see also Fig. 8 of the main text) and the areas were identified by transferring the respective masks to the corresponding morphology images.

2. Crystal Structures of HTMs on different double perovskite substrates

Structural analysis of the evaporated hole transport layers was conducted by means of X-ray diffraction (XRD). For CuPc, this was further extended by *in-situ* UV-VIS absorption spectroscopy during film growth.

2.1 Formation of different CuPc polymorphs

Fig. S11 depicts the XRD of CuPc evaporated on pristine and modified $Cs_2AgBiBr_6$, as well as on $BA_4AgBiBr_8$ and $PEA_4AgBiBr_8$ double perovskite thin films, respectively, using the same samples analyzed in the main text. For pristine and BABr-modified $Cs_2AgBiBr_6$, formation of the α-phase can be confirmed by the expected signal for the (200)-plane around 6.83 - 6.86°.[5–7] The significantly higher intensity of the signal on BABr-modified $Cs_2AgBiBr_6$ points towards an enhanced crystalline order of CuPc compared to that grown on pristine $Cs_2AgBiBr_6$. Although the signal of CuPc on $BA_4AgBiBr_8$ is superimposed to dominating reflections of the latter, the asymmetry of the peak might hint at formation of crystalline CuPc in the α- or possibly η-phase. On $PEA_4AgBiBr_8$, a characteristically different diffractogram was found, in which the η-phase of CuPc was clearly identified by signals at 6.95° and 8.6°, assigned to the (001)- and $(20\bar{1})$-planes, respectively.[1,8] For CuPc on PEABr-modified $Cs_2AgBiBr_6$, the same two signals of the η-phase were observed. In Fig. S11, vertical lines further clarify the presence

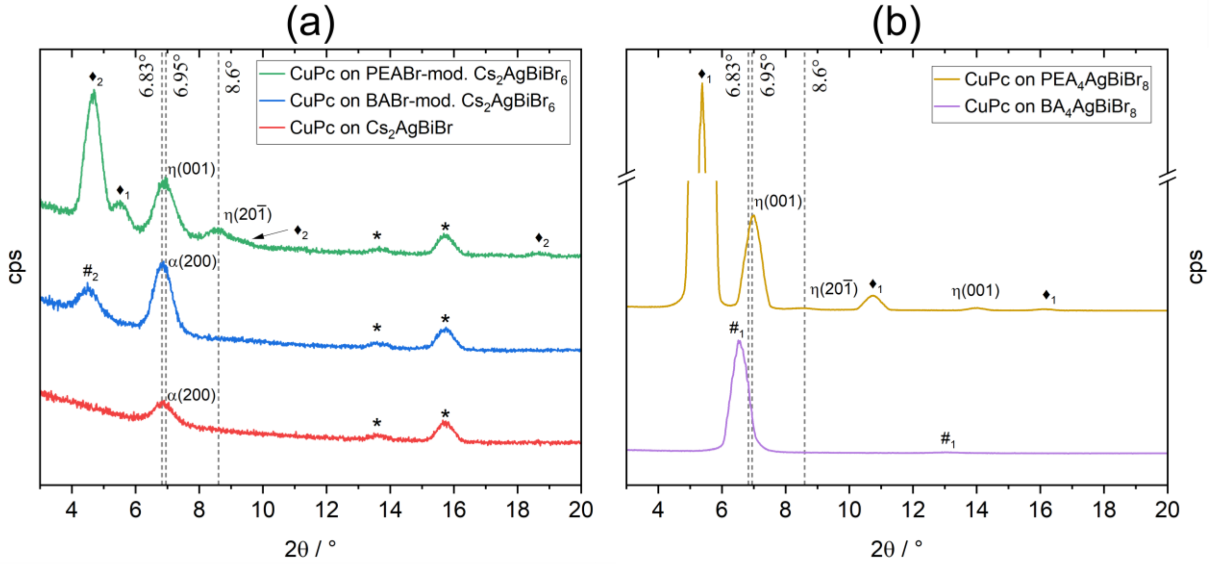

Fig. S11. GI-XRD measurements of 90 nm CuPc evaporated on pristine and modified $Cs_2AgBiBr_6$ (a), as well as on $BA_4AgBiBr_8$ and $PEA_4AgBiBr_8$ (b) thin films. Signals assigned to the different lattice directions of α or η crystal phases of CuPc are explicitly shown. Those corresponding to $Cs_2AgBiBr_6$ are marked by (*), to 2D BA- or 2D PEA layered phases by ($\#_n$), or ($\blacklozenge_n$), respectively, with the subscript n indicating the number of octahedral layers between the spacer cations of the respective 2D phase.

of distinct signals for the α- and η-phases on the different substrates. Despite a very similar position of the η(001) and α(200) signals, the peaks are tentatively assigned to a given polymorph based on their detailed position.

The systematic differences in the crystal structure of CuPc on the different samples correlates with the different film morphology of CuPc found in the main text. Samples that exhibited long needles at low CuPc film thickness consistently gave XRD-signals corresponding to the η-phase, while short needles gave an XRD-signal corresponding to the α-phase. To further analyze the nucleation of CuPc during film growth *in-situ* UV-VIS transmission measurements were conducted. Although a slightly different sample geometry and a different vacuum chamber were used for these experiments, conformity with the other samples was ensured by XRD and UV-VIS measurements on both sets of samples. Fig. S12 illustrates the evolution of the absorption

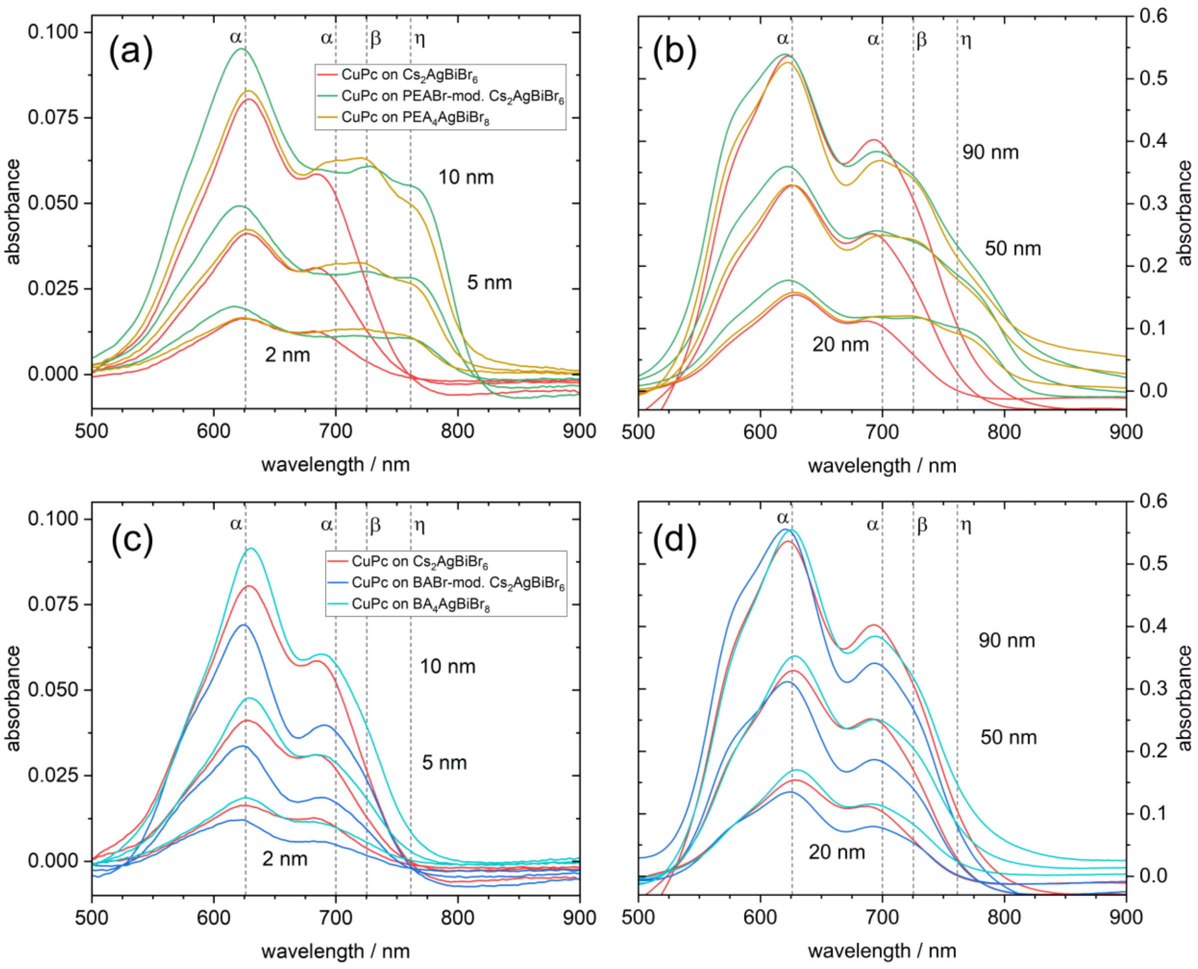

Fig. S12. UV-VIS absorption spectra measured in transmission at different average film thickness of CuPc as indicated during deposition on pristine $Cs_2AgBiBr_6$ compared to PEABr-modification and $PEA_4AgBiBr_8$ ((a)-(b)), and to BABr-modification and $BA_4AgBiBr_8$ ((c)-(d)). Approximate peak positions were taken from literature.[1,8]

spectra of CuPc evaporated on pristine, PEABr- and BABr-modified $Cs_2AgBiBr_6$, as well as on $PEA_4AgBiBr_8$ and $BA_4AgBiBr_8$ with increasing average CuPc film thickness. Already at 2 nm, the spectra of CuPc on PEABr-modified $Cs_2AgBiBr_6$ and $PEA_4AgBiBr_8$ (Fig. S12 (a)) significantly differed compared to pristine $Cs_2AgBiBr_6$ and BABr-containing samples (Fig. S12 (c)). While the latter only showed contributions arising from the α-phase around 625 nm[1,5,8,9] and 700 nm,[1,5,8,9] the other two showed significant contributions assigned to the β- and η-phases at around 725 nm[1,8] and 770 nm,[1,8] respectively. The relative intensity of the signals at 700 nm, 725 nm and 770 nm stayed widely constant up to 20 nm (Fig. S12 (b)), from where on the α- and β-phases of CuPc obviously started to dominate over the η-phase also for the PEA-containing samples. Although up to 90 nm average film thickness, all three phases could be identified, the spectra indicate that between 50 nm and 90 nm, nucleation primarily took place in the α- and β-phases since the contribution of the η-phase to the spectra did not change significantly compared to the α- and β-phases. This predominance of the α- and β-phases came along with a shortening of the needles, which then were of medium length only. But since the η-phase still provided the only signal of CuPc in the XRD (Fig. S11), the crystal size or quality must be considerably higher compared to crystals in the α- or β-phases. A correlation between the length of the observed needles and the portion of η-CuPc is, therefore, indicated. On pristine $Cs_2AgBiBr_6$ and BABr-containing samples, following formation of the α-phase at low film thickness, the α-phase continued to grow, just accompanied by a small shoulder characteristic of the β-phase starting to contribute between 20 nm and 50 nm. This supports that the short needles present on $BA_4AgBiBr_8$ would consist of the α-phase and, hence, the relation between morphology and crystal phases is confirmed. Accordingly, the α-phase occurred in short needles on both $BA_4AgBiBr_8$ and PEA-containing samples, whereas the η-phase occurred in long needles on the PEA-containing samples.

In summary, CuPc nucleated simultaneously in the α-, β- and η-phases on PEABr-modified $Cs_2AgBiBr_6$ and on $PEA_4AgBiBr_8$, with decreasing relative amounts of the η-phase at higher film thickness. Mainly the α-phase was formed on pristine and BABr-modified $Cs_2AgBiBr_6$ and $BA_4AgBiBr_8$. Despite differences in the observed sample morphology and work function, the BA^+ spacer cation did not affect the crystal structure of CuPc during its growth, in contrast to PEA^+, which led to a characteristically different type of CuPc growth, namely long needles in the η-phase. Nevertheless, the flat layered terraces of $BA_4AgBiBr_8$ as well induced formation of CuPc needles, albeit shorter and of the α-phase.

2.2 Formation of different Pn phases

XRD of Pn on the different substrates is shown in Fig. S13. While the expected signal of the thin film phase of Pn could be clearly identified on $BA_4AgBiBr_8$, it is superimposed to the main signal of $PEA_4AgBiBr_8$. For the sample on $PEA_4AgBiBr_8$, additional signals at 6.9° and the corresponding second order at 13.8° were detected. These are assigned to formation of an undesired side phase of $Ag_3Bi_{14}Br_{21}$ [10] formed during preparation. Its presence is discussed below. On pristine, BABr-modified and PEABr-modified $Cs_2AgBiBr_6$, a widely identical phase composition was found. It is characterized by a rather broad symmetric peak at 5.9° accommodating the signals of the thin film and bulk phases at 5.7° and 6.1°,[2–4] respectively. Since the peak position did not vary, the ratio of the two phases seemed to be constant for all three substrates. On BABr- and PEABr-modified $Cs_2AgBiBr_6$, weak signals of second order were seen at 11.4° for the thin film and 12.2° for the bulk phase, respectively, which confirmed the assignment of the signal at 5.9°.

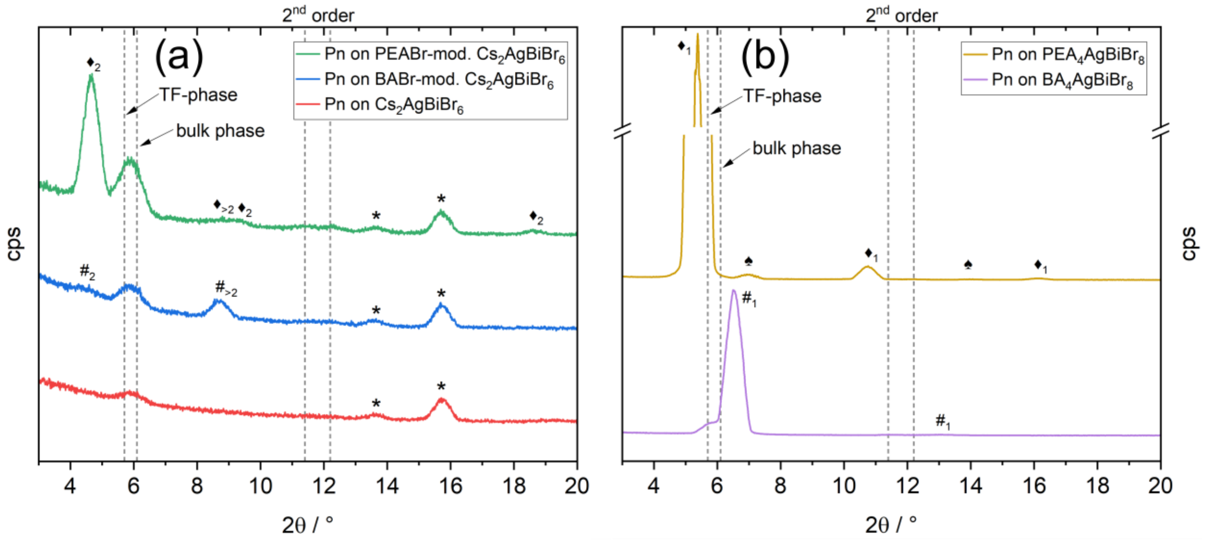

Fig. S13. GI-XRD measurements of 90 nm Pn evaporated on pristine and modified $Cs_2AgBiBr_6$ (a), as well as on $BA_4AgBiBr_8$ and $PEA_4AgBiBr_8$ (b) thin films. Signals corresponding to $Cs_2AgBiBr_6$ are marked by (*), 2D BA layered phases by ($\#_n$), 2D PEA layered phases by ($♦_n$) and $Ag_3Bi_{14}Br_{21}$ by (♠). The subscript n indicates the number of octahedral layers between the spacer cations of the respective 2D phase.

3. Side phase in $PEA_4AgBiBr_8$ thin films following solvent engineering

A solvent engineering approach by using a mixture of 80% DMF and 20% DMSO as solvent led to the desired large crystalline domains of $PEA_4AgBiBr_8$. However, it led to the formation of an undesired side phase of $Ag_3Bi_{14}Br_{21}$, also (Fig S13 (b)). Further optimization of the preparation routine could not prevent this. The phase was identified by signals at 6.9° and 13.8° in XRD (Fig. S14 (a)) in addition to the signals expected for $PEA_4AgBiBr_8$.[11] Height images obtained by confocal laser microscopy revealed that such contaminated films featured macroscopic crystalline filaments sticking out of the surface (Fig. S14 (b)), whereas no such features were found on a pure $PEA_4AgBiBr_8$ film prepared by the conventional method[11] with only DMF as solvent, without signals of $Ag_3Bi_{14}Br_{21}$ in XRD (Fig. S14 (c)). Further, the intensity of the $Ag_3Bi_{14}Br_{21}$ XRD-signals was found proportional to the number of filaments present on the sample. Therefore, the growth studies in this work were performed on those large parts of the engineered $PEA_4AgBiBr_8$ films that were free of such filaments and, hence, consisted of phase-pure $PEA_4AgBiBr_8$ without any contamination by $Ag_3Bi_{14}Br_{21}$. This side phase, however, was superimposed to the signal of the CuPc η(001)-plane in XRD of CuPc on $PEA_4AgBiBr_8$ in Fig. S11 (b). Nevertheless, the η-structure of CuPc could be successfully identified because of a significantly increased signal intensity compared to the diffractogram of the solvent-engineered $PEA_4AgBiBr_8$ as seen in, e.g., Fig. S13 (b) and was independently confirmed by UV-Vis spectroscopy (Fig. S12 (a) and (b)).

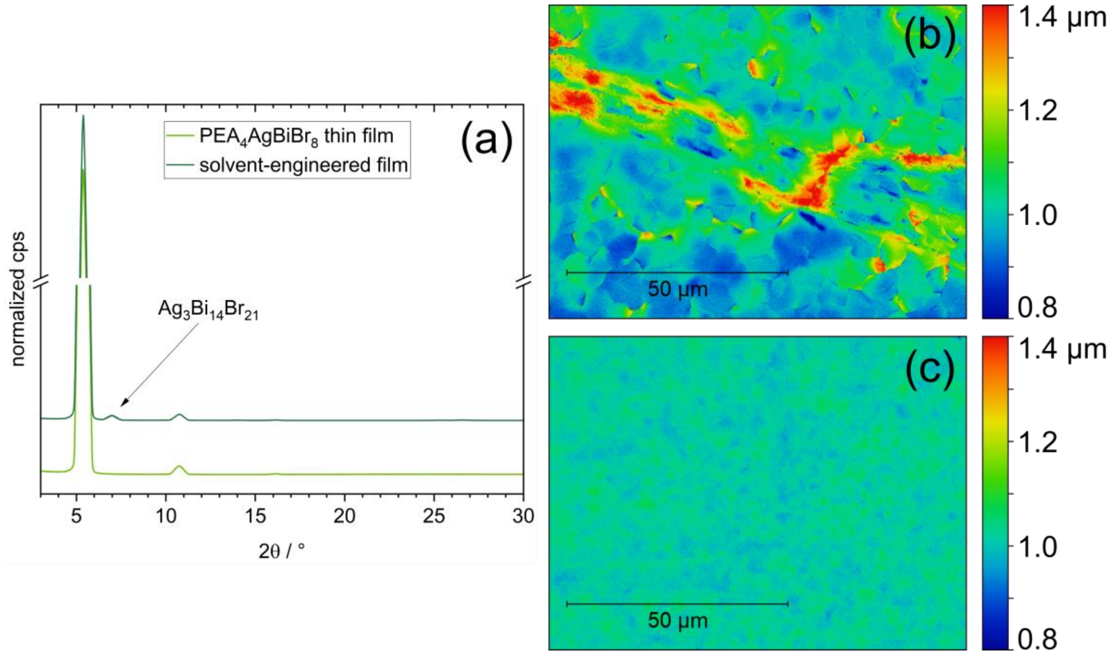

Fig. S14. GI-XRD measurements of a phase-pure and contaminated $PEA_4AgBiBr_8$ thin film (a), and microscope images (150-times magnification) of $Ag_3Bi_{14}Br_{21}$ contaminations (b) and film without contamination (c).

4. Stability of photovoltaic devices

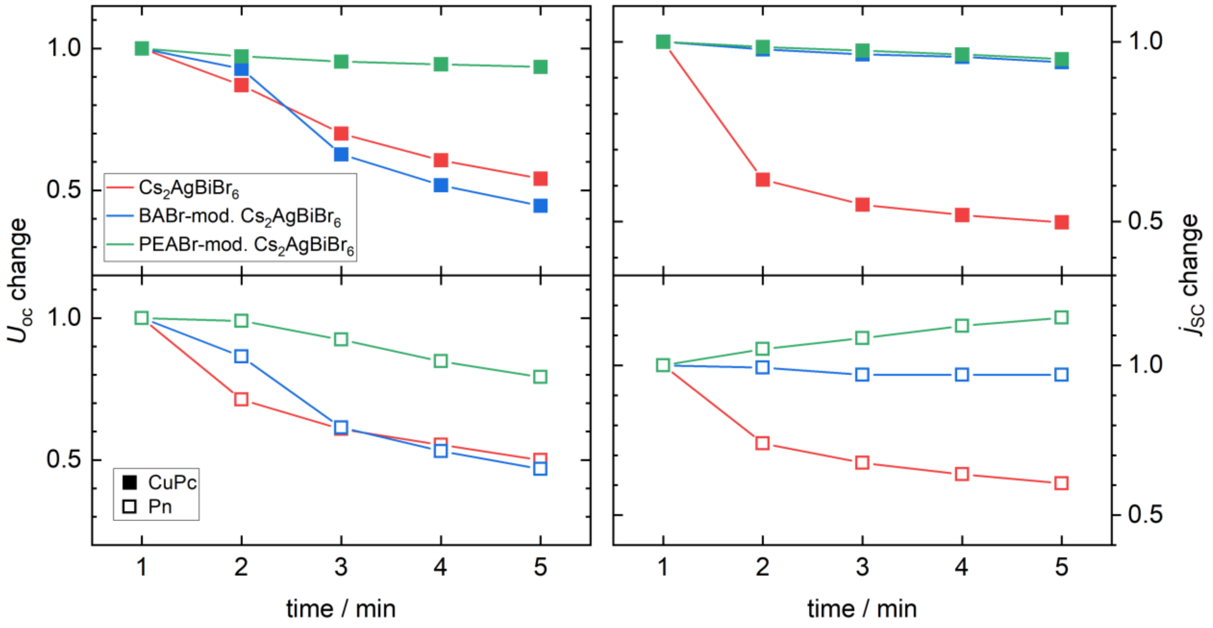

Fig. S15. Stability of the open-circuit potential U_{OC} (left) and short-circuit current density j_{SC} (right) over time for FTO/TiO_2/(pristine or 2D-modified) $Cs_2AgBiBr_6$/HTM/Au devices with vapor-deposited CuPc (filled squares) or Pn (empty squares) as HTM.

5. References

(1) Wang, H.; Mauthoor, S.; Din, S.; Gardener, J. A.; Chang, R.; Warner, M.; Aeppli, G.; McComb, D. W.; Ryan, M. P.; Wu, W.; Fisher, A. J.; Stoneham, M.; Heutz, S. Ultralong copper phthalocyanine nanowires with new crystal structure and broad optical absorption. *ACS nano* **2010**, *4* (7), 3921–3926. DOI: 10.1021/nn100782w.

(2) Bouchoms, I.; Schoonveld, W. A.; Vrijmoeth, J.; Klapwijk, T. M. Morphology identification of the thin film phases of vacuum evaporated pentacene on SiO2 substrates. *Synth. Met.* **1999**, *104* (3), 175–178. DOI: 10.1016/S0379-6779(99)00050-8.

(3) Gundlach, D. J.; Jackson, T. N.; Schlom, D. G.; Nelson, S. F. Solvent-induced phase transition in thermally evaporated pentacene films. *Appl. Phys. Lett.* **1999**, *74* (22), 3302–3304. DOI: 10.1063/1.123325.

(4) Dramstad, T. A.; Wu, Z.; Gretz, G. M.; Massari, A. M. Thin Films and Bulk Phases Conucleate at the Interfaces of Pentacene Thin Films. *J. Phys. Chem. C* **2021**, *125* (30), 16803–16809. DOI: 10.1021/acs.jpcc.1c04432.

(5) Ai, X.; Lin, J.; Chang, Y.; Zhou, L.; Zhang, X.; Qin, G. Phase modification of copper phthalocyanine semiconductor by converting powder to thin film. *Appl. Surf. Sci.* **2018**, *428*, 788–792. DOI: 10.1016/j.apsusc.2017.09.146.

(6) Lozzi, L.; Santucci, S.; La Rosa, S.; Delley, B.; Picozzi, S. Electronic structure of crystalline copper phthalocyanine. *J. Chem. Phys.* **2004**, *121* (4), 1883–1889. DOI: 10.1063/1.1766295.

(7) Prabakaran, R.; Kesavamoorthy, R.; Reddy, G.; Xavier, F. P. Structural Investigation of Copper Phthalocyanine Thin Films Using X-Ray Diffraction, Raman Scattering and Optical Absorption Measurements. *phys. stat. sol. (b)* **2002**, *229* (3), 1175–1186. DOI: 10.1002/1521-3951(200202)229:3<1175:AID-PSSB1175>3.0.CO;2-K.

(8) Zou, T.; Wang, X.; Ju, H.; Zhao, L.; Guo, T.; Wu, W.; Wang, H. Controllable Molecular Packing Motif and Overlap Type in Organic Nanomaterials for Advanced Optical Properties. *Crystals* **2018**, *8* (1), 22. DOI: 10.3390/cryst8010022.

(9) Farag, A. Optical absorption studies of copper phthalocyanine thin films. *Opt. Laser Technol.* **2007**, *39* (4), 728–732. DOI: 10.1016/j.optlastec.2006.03.011.

(10) Wahl, B.; Ruck, M. Ag3Bi14Br21 : ein Subbromid mit Bi24+-Hanteln und Bi95+-Polyedern – Synthese, Kristallstruktur und Chemische Bindung. *Z. fur Anorg. Allg. Chem.* **2008**, *634* (15), 2873–2879. DOI: 10.1002/zaac.200800320.

(11) Schmitz, F.; Horn, J.; Dengo, N.; Sedykh, A. E.; Becker, J.; Maiworm, E.; Bélteky, P.; Kukovecz, Á.; Gross, S.; Lamberti, F.; Müller-Buschbaum, K.; Schlettwein, D.; Meggiolaro,

D.; Righetto, M.; Gatti, T. Large Cation Engineering in Two-Dimensional Silver–Bismuth Bromide Double Perovskites. *Chem. Mater.* **2021**, *33* (12), 4688–4700. DOI: 10.1021/acs.chemmater.1c01182.
